# Integrative morphological, biochemical, and plastome-based characterization of a wild plum (*Prunus* spp.) population from Mount Erciyes (Central Anatolia, Türkiye)

**DOI:** 10.1186/s40659-026-00702-0

**Published:** 2026-05-27

**Authors:** Samuel Obedgiu, Kahraman Gürcan, Nurdan Tuna Güneş, Ahmet Sümbül, Mehmet Yaman, Vahid Roumi, Yazgan Tunç

**Affiliations:** 1 Department of Agricultural Biotechnology, Institute of Natural Sciences, Erciyes University, 38039 Kayseri, Türkiye; 2 Betul Ziya Eren Genome and Stem Cell Center, Erciyes University, 38280 Kayseri, Türkiye; 3 Department of Agricultural Biotechnology, Faculty of Agriculture, Erciyes University, 38280 Kayseri, Türkiye; 4 Department of Horticulture, Faculty of Agriculture, Ankara University, 60110 Ankara, Türkiye; 5 Department of Plant and Animal Production, Suşehri Timur Karabal Vocational School, Sivas Cumhuriyet University, 58600 Sivas, Türkiye; 6 Department of Horticulture, Faculty of Agriculture, Erciyes University, 38030 Kayseri, Türkiye; 7 Plant Protection Department, Faculty of Agriculture, University of Maragheh, Maragheh, 55187 Iran; 8 Republic of Türkiye, Ministry of Agriculture and Forestry, General Directorate of Agricultural Research and Policies, Hatay Olive Research Institute Directorate, Hassa Station, 31700 Hassa, Hatay Türkiye

**Keywords:** *Prunus* spp., Plastome sequencing, Phenotypic diversity, Phylogenetic analysis, Wild germplasm

## Abstract

Plums (*Prunus* spp.) are among the most diverse fruit tree species, yet their wild relatives remain poorly characterized despite their importance for breeding, conservation, and understanding evolutionary diversity. Here, we investigated a wild plum population from Mount Erciyes, located in Central Anatolia, Türkiye, through integrated morphological, textural, total content-based biochemical, and plastome analyses. Floral morphology of the Erciyes wild plums displayed intermediate features between apricot (*P. armeniaca*) and cultivated plum (*P. domestica*), while fruit texture exhibited significantly higher peel hardness and flesh resistance compared with the reference cultivar ‘Papaz Eriği’. Illumina sequencing of the chloroplast genome produced a 157,840 bp plastome with a conserved quadripartite structure, encoding 125 genes. Comparative analyses revealed minor structural differences in the LSC and SSC regions, whereas the IR regions remained highly conserved. Phylogenetic reconstruction based on complete plastomes clustered the Erciyes wild plums with *P. domestica* and *P. cerasifera*, reflecting plastome-level phylogenetic affinity. This study identified significant phenotypic and total content-based biochemical diversity in wild plum genotypes. Key traits such as fruit and seed weight, color, and bioactive compound contents showed wide variability. Based on the heatmap hierarchical clustering analysis, the genotypes and the examined traits were divided into two main groups exhibiting significant positive and negative correlations among the traits. Together, these findings provide a characterization of a previously undocumented wild plum from Mount Erciyes and establish a baseline reference for future studies on plum evolution, conservation, and breeding.

**Supplementary Information:**

The online version contains supplementary material available at 10.1186/s40659-026-00702-0.

## Introduction

Plums (*Prunus* spp., Rosaceae) represent one of the most diverse groups of fruit trees, with their common ancestor estimated to have evolved around 31 million years ago [1, 2]. They display remarkable variation in fruit size, shape, flavor, aroma, texture, and color, making them highly valued since ancient times. Among the numerous species, *P. domestica*, *P. salicina*, and *P. simonii* were domesticated early in human history [3]. Vavilov [4] proposed that the center of origin of *P. domestica* lies around the Caspian Sea and the Caucasus, where *P. cerasifera* and *P. spinosa* are distributed. In contrast, *P. salicina* and *P. simonii* were introduced and cultivated in Asia [5]. Despite broad genetic and morphological diversity, cultivated plums are mainly divided into two groups: diploid Japanese plum (*P. salicina*, 2n = 2x = 16) and hexaploid European plum (*P. domestica*, 2n = 6x = 48). According to fruit use, plums are typically consumed fresh (*P. salicina* and hybrids) or dried (*P. domestica*) [6]. Accordingly, this study aims to provide an integrated characterization of a wild plum from Mount Erciyes by combining phenotypic traits (floral morphology and fruit texture) with total phenolic content (TPC), total flavonoid content (TFC), total anthocyanin content (TAC), as well as antioxidant activity (AA), together with plastome-level genomic analysis. Rather than inferring domestication processes directly, we use plastome phylogeny to place this wild plum within the European plum lineage and to contextualize observed phenotypic diversity.

Türkiye plays a central role in global plum cultivation, ranking third worldwide among stone fruits after olives and peaches. The country produced 348,750 tons of plums in 2022, with production concentrated in the Mediterranean, Marmara, and Aegean regions. Major provinces include Mersin, Bursa, Adana, Izmir, Antalya, and Manisa [7]. Anatolia is also recognized as a center of genetic diversity for *P. cerasifera*, *P. insititia*, *P. spinosa*, and *P. domestica* [8].

The chloroplast genome offers valuable insights into plant systematics and evolution due to its haploid nature, conserved structure, and uniparental inheritance. Since the sequencing of the tobacco chloroplast genome in 1986 [9], advances in sequencing technologies have facilitated plastome studies across angiosperms [10]. Chloroplast genomes of many *Prunus* species have now been reported, including *P. persica* [11], *P. mume* [12], *P. tomentosa* [13], *P. cerasoides* [14], *P. domestica* [15], and *P. salicina* [16, 17]. These studies reveal highly conserved quadripartite structures but also highlight species-specific variations. However, most research has focused on domesticated plums, leaving wild relatives poorly characterized. This gap limits the use of wild plums as genetic resources for breeding and conservation.

Beyond genetics, fruit texture, TPC, TFC, TAC, and AA are critical determinants of fruit quality, consumer acceptance, and storage potential [18, 19]. Wild plums often contain high levels of phenolics, antioxidants, and other metabolites linked to human health benefits, including reduced risk of chronic diseases such as diabetes and coronary heart disease [20–22]. Despite their nutritional potential, wild plums remain underexplored.

Mount Erciyes in Central Anatolia represents an important center of plant diversity, harboring wild *Prunus* trees among oak and aspen stands. The flowers and fruits of this population resemble plums, though young leaves are apricot-like, and mature tree trunks resemble almonds. Fruits mature in late September are typically hard and astringent, yet possess a strong fragrance and are traditionally used in local medicinal practices. Given its geographic position near regions historically discussed in relation to plum diversity, and its unique morphological traits, the Erciyes wild plum provides an exceptional opportunity to document and characterize wild plum diversity and to identify traits of potential relevance for future evolutionary and breeding studies. This study aims to sequence and characterize the chloroplast genome of Erciyes wild plums to establish their plastome structure and to place this wild plum within the broader phylogenetic framework of *Prunus*. Furthermore, it analyzes TPC, TFC, TAC, and AA parameters and evaluates fruit texture and morphological traits, to explore evolutionary patterns and their associations with genetic and phenotypic data. Rather than addressing these components as independent objectives, the present study integrates phenotypic, total content-based biochemical, and plastome-level analyses to provide a coherent characterization of a previously undocumented wild plum. The plastome analysis establishes phylogenetic context, while morphological, textural, and TPC, TFC, TAC, and AA describe functional and phenotypic diversity within that framework.

This study aims to determine whether the Erciyes wild plum represents a distinct phenotypic profile, including variation in TPC, TFC, TAC, and AA, within the European plum lineage and to assess the extent of variability associated with plastome-based phylogenetic affinity.

## Materials and methods

### Plant material

**Fig. 1:**
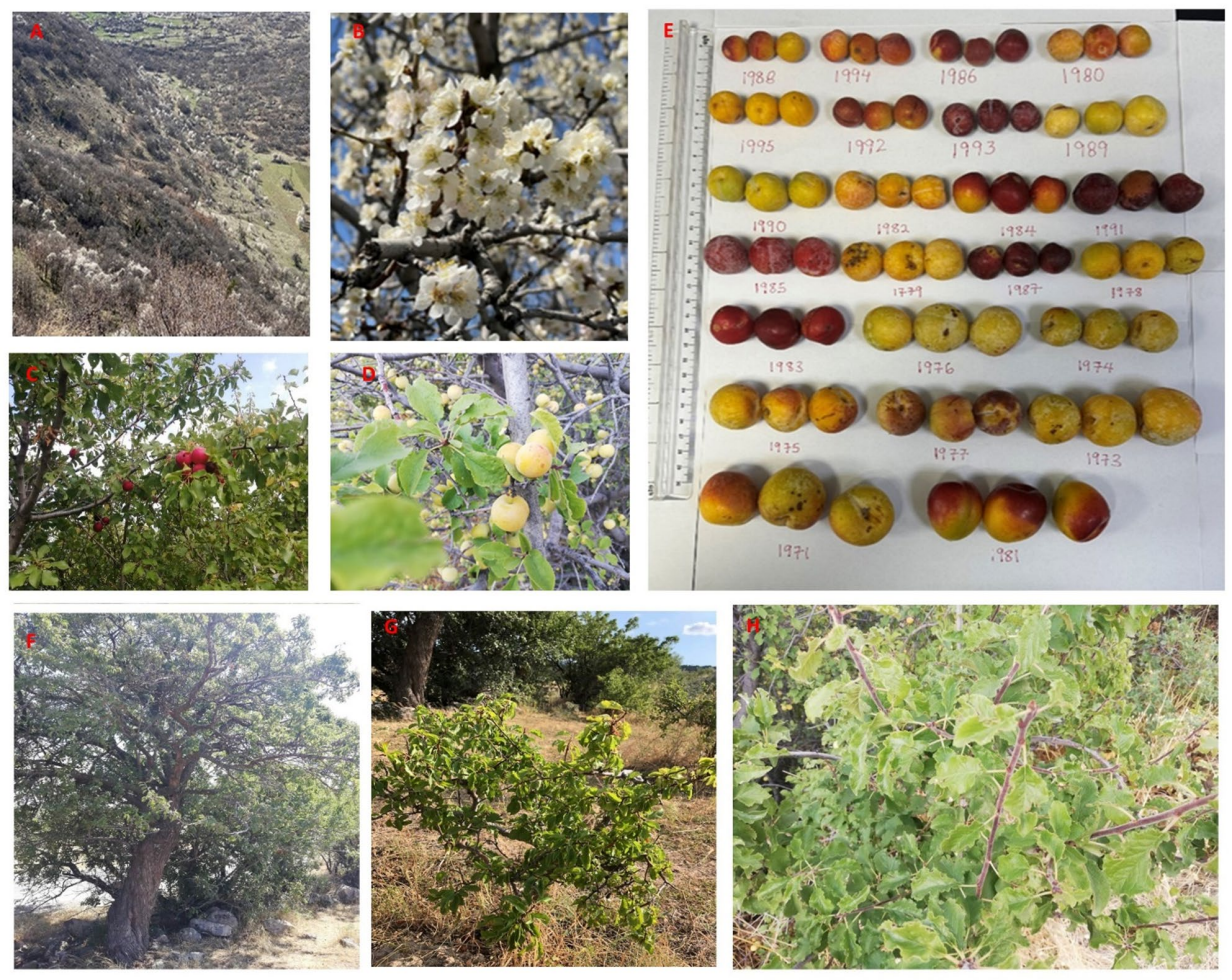
Morphological characteristics of wild plum trees from Mount Erciyes, Türkiye. **A** Flowering wild plum trees growing on the slopes of Mount Erciyes among oak stands, illustrating their natural habitat. **B** Close-up view of flowers. **C**–**E** Fruits showing variation in color and shape. **F** Mature tree exhibiting trunk morphology similar to that of almond (*P. dulcis*). **G** Young tree. **H** Leaf of a young tree, morphologically resembling apricot leaves, highlighting age-related developmental variation. This photograph was captured as part of the present study and was provided by the second author, Prof. Dr. Kahraman Gürcan. It is not subject to any third-party copyright restrictions

A total of 24 wild plum trees located on Mount Erciyes in Central Anatolia, Türkiye, were randomly selected for the evaluation of floral morphology, fruit texture, and TPC, TFC, TAC, and AA (Fig. 1). The sampling was carried out along the mountainous transect extending from Kayseri to Kızıkören village, covering the area between 38.64509° N, 35.39151° E and 38.64054° N, 35.36122° E. For plastome sequencing, two representative trees were selected based on fruit color variation, one bearing yellow fruits and the other red (Fig. 1C, D). These two individuals were selected to represent contrasting phenotypic types (red- and yellow-fruited genotypes) in order to evaluate potential plastome-level differences. Plastome sequencing was conducted to infer maternal phylogenetic placement rather than to assess intra-population chloroplast diversity, and therefore a limited number of representative samples was considered sufficient for this purpose. In addition, for comparative floral analyses, samples were collected from a local apricot cultivar (*Prunus armeniaca* ‘Kayseri Pa’) and a cultivated plum (*P. domestica* ‘Papaz Eriği’). Taxonomic identification of all plant materials was performed by Prof. Dr. Kahraman Gürcan. Although slight variation in ripening time was observed among genotypes, all fruits were harvested at a comparable physiological maturity stage corresponding to the onset of commercial ripeness. This stage was determined based on local empirical criteria, including fruit color development and ease of detachment, as recognized by residents of the sampling area. This approach minimized potential bias associated with maturity differences in fruit texture measurements.

### The characteristics evaluated

#### Floral morphology

Fresh flowers were collected during the spring season and analyzed immediately after harvest. Floral traits were recorded through direct observation and manual measurements using a standard ruler. Morphological characteristics, including petal and sepal dimensions, coloration, and texture, were systematically evaluated. Pistil length and stigma morphology were measured to characterize the gynoecium, whereas stamen number and filament length were recorded to describe the androecium. Six flowers per species were analyzed to account for intraspecific variation.

#### Fruit texture analysis

Fruit texture properties were determined using 8–25 fruits per genotype. Fruits of the plum cultivar ‘Angelic’ were included as a control. Measurements were taken from opposite sides of each fruit using a texture analyzer (TA.XT2, Stable Micro Systems, UK) equipped with a 50 kg load cell and an SMS P/2 (Ø 2 mm) probe. Data acquisition and processing were performed using Exponent TE32 software (v5.06.0). The puncture test was conducted under the following conditions: pre-test speed of 10 mm s^−1^, test speed of 10 mm s^−1^, post-test speed of 10 mm s^−1^, target distance of 4.0 mm, auto force trigger mode, and a trigger force of 10 g. The extracted texture parameters included peel hardness (N), peel elasticity (mm), energy absorption capacity (mJ), brittleness (N mm^−1^), resistance of flesh to force (mJ), and flesh firmness (N).

#### Fruit morphology analysis

To evaluate phenotypic diversity among plum genotypes, 30 fruits were randomly selected and subjected to morphological measurements. Fruit and seed weights were determined using a precision balance (Radwag AS 220.R2, Radwag, Poland) with a sensitivity of ± 0.01 g. Fruit traits, including fruit width, length, and flesh thickness, were measured using a digital caliper (Mitutoyo 500-196-30, Mitutoyo Corp., Japan) with a resolution of 0.01 mm. Peel color was determined using a colorimeter (Minolta CR-400, Konica Minolta, Japan), and color parameters were recorded in the CIELAB color space (L*, a*, b*).

#### Total content-based biochemical composition of fruits

For biochemical analyses, plum fruits (including peel and flesh) were homogenized after removal of the stones. A 5 g portion of the homogenized sample was weighed using a precision balance (Radwag AS 220.R2, Radwag, Poland), and 25 mL of methanol (HPLC grade) was added. The mixture was homogenized for 2 min using a vortex mixer (Vortex-Genie 2, Scientific Industries, USA) and subsequently incubated in the dark at + 4 °C for 16 h. The samples were then centrifuged at 10,000 rpm for 20 min using a refrigerated centrifuge (Hettich Universal 320R, Andreas Hettich GmbH, Germany). The resulting supernatant was carefully collected using a micropipette (Eppendorf Research Plus, Eppendorf, Germany) and transferred into Falcon tubes for further analyses.

*Total phenolic content (TPC)* The total phenolic content (TPC) of plum genotypes was determined by a modified Folin–Ciocalteu method, as described by Singleton and Rossi [23]. Briefly, 500 µL of fresh fruit extract was mixed with 4.1 mL of distilled water, 100 µL of Folin–Ciocalteu reagent (Sigma-Aldrich, USA), and 2% sodium carbonate (Na_2_CO_3_). The mixture was incubated in the dark for 2 h at room temperature. Absorbance was measured at 760 nm using a UV–Vis spectrophotometer (Shimadzu UV-1800, Shimadzu Corporation, Japan). Results were expressed as mg gallic acid equivalents (GAE) per 100 g fresh weight [24].

*Total flavonoid content (TFC)* Total flavonoid content (TFC) was determined according to Demir et al. [25]. One mL of fruit extract was mixed with 3.3 mL of methanol, followed by the addition of 0.1 mL of 10% AlCl_3_·6 H_2_O and CH_3_COOK solution. The mixture was vortexed using a vortex mixer (Vortex-Genie 2, Scientific Industries, USA) and incubated at room temperature. Absorbance was measured at 415 nm using a UV–Vis spectrophotometer (Shimadzu UV-1800, Shimadzu Corporation, Japan). Flavonoid content was calculated as quercetin equivalents (QE) and expressed as mg QE/100 g.

*Antioxidant activity (AA)* Antioxidant activity (AA) was determined by a modified DPPH method developed by Brand-Williams et al. [26]. For each genotype, 100 µL of fruit extract was mixed with 2.9 mL of ethanol and 1 mL of DPPH solution (0.26 mM 1,1-diphenyl-2-picrylhydrazyl), followed by vortex mixing. The mixture was incubated in the dark for 30 min at room temperature. Absorbance was measured at 517 nm using a UV–Vis spectrophotometer (Shimadzu UV-1800, Shimadzu Corporation, Japan). Antioxidant activity was expressed as percentage inhibition.

*Total anthocyanin content (TAC)* Total anthocyanin content (TAC) was determined using the pH differential method described by Giusti and Wrolstad [27]. Samples were diluted with buffer solutions at pH 1.0 and pH 4.5 and incubated for 30 min. Absorbance was measured at 527 and 700 nm using a UV–Vis spectrophotometer (Shimadzu UV-1800, Shimadzu Corporation, Japan). Results were calculated as cyanidin-3-glucoside equivalents and expressed as mg cyanidin-3-glucoside/100 g.

All total content-based biochemical analyses were performed using three technical replicates for each genotype, and the results are presented as mean ± standard deviation. Calibration curves were constructed using appropriate standards, including gallic acid for total phenolic content and quercetin for total flavonoid content. All results were expressed on a fresh weight (FW) basis. Calibration curves were constructed using standard solutions, and showed high linearity (R^2^ > 0.99).

#### Chloroplast genome sequencing and annotation

Total genomic DNA was extracted from fresh young leaves using a modified CTAB method [28]. Sequencing libraries with an average insert size of ~ 350 bp were prepared and sequenced on the Illumina HiSeq 4000 platform (150 bp paired-end reads; Macrogen, Daejeon, Korea). De novo assembly of the plastome was performed, and annotation was carried out using the CPGAVAS2 and GeSeq pipelines [29, 30]. Transfer RNA genes were identified using tRNAscan-SE [31], and circular plastome maps were generated with OGDraw v1.3.1 [32]. Contraction and expansion at the junctions of the LSC, SSC, IRa, and IRb regions were analyzed using IRscope [33]. Phylogenetic analysis was conducted using the maximum likelihood method implemented in MEGA12 [34], with 1000 bootstrap replicates.

Given the highly conserved nature of plastid genomes in *Prunus* and their limited variation within local populations, plastome sequencing was not intended to capture intra-population diversity but rather to establish phylogenetic context at the maternal lineage level.

### Statistical analysis

All analyses were conducted using three replications per genotype, with measurements performed on 10 randomly selected fruits within each replication. Mean values and coefficients of variation (CV) were calculated to assess variability in morphological traits and in TPC, TFC, TAC, and AA. Variance analysis (ANOVA), principal component analysis (PCA), heatmap hierarchical clustering, and correlation analyses were performed using JMP^®^ Pro 17.0 software (SAS Institute Inc., Cary, NC, USA). Differences among mean values were evaluated using Tukey’s multiple comparison test at a significance level of *p* < 0.05. Multivariate statistical analyses were employed to investigate relationships among the examined traits. Principal component analysis (PCA) was used to determine the contribution of variables and to evaluate relationships among genotypes. Kaiser normalization and Varimax rotation were applied to enhance the interpretability of the principal components and to facilitate the identification of trait–component loadings [35, 36]. Components with eigenvalues ≥ 1 were retained according to Kaiser’s criterion [35, 36]. A biplot based on the first two principal components (PC1 and PC2) was constructed to visualize the distribution of genotypes and associated traits. Heatmap hierarchical clustering was performed using the Ward method based on Euclidean distance to classify genotypes and traits and to provide a comprehensive visualization of their relationships [37]. Correlation matrix analysis was conducted using OriginPro 2025 software (OriginLab Corporation, Northampton, MA, USA) to determine the direction and magnitude of relationships among the examined traits. Pearson correlation coefficients (r), which are sensitive to linear associations, were calculated to identify positive and negative interactions between variables [38].

## Results

### Floral morphology

Flowers of the Erciyes wild plum exhibited predominantly white petals, comparable to those of the apricot cultivar ‘Kayseri Pa’ and the cultivated plum ‘Papaz Eriği’, although minor differences in size were observed (Table 1 and Supplementary Tables S1–S5). Sepals of the wild plum were green, resembling those of ‘Papaz Eriği’, whereas ‘Kayseri Pa’ exhibited maroon sepals (Supplementary Table S1). Quantitative comparisons revealed significant interspecific differences. Sepal length and width were highest in ‘Papaz Eriği’ (0.60 ± 0.08a cm and 0.40 ± 0.04a cm, respectively), intermediate in the wild plum (0.33 ± 0.03b cm and 0.25 ± 0.04b cm), and lowest in ‘Kayseri Pa’ (0.33 ± 0.03b cm and 0.16 ± 0.05c cm). A similar trend was observed for style length, with the longest styles in ‘Papaz Eriği’ (1.00 ± 0.14a cm), followed by the wild plum (0.90 ± 0.08a cm), and the shortest in ‘Kayseri Pa’ (0.65 ± 0.13b cm) (Supplementary Table S2). Ovary dimensions were also significantly greater in ‘Papaz Eriği’ than in both the wild plum and apricot. In contrast, pedicel length was markedly greater in ‘Kayseri Pa’ (0.88 ± 0.10a cm) compared with both the wild plum and ‘Papaz Eriği’ (0.33 ± 0.06b cm and 0.35 ± 0.05b cm, respectively) (Supplementary Table S3). Stamen characteristics also differed significantly among taxa. The wild plum exhibited the longest filaments (0.61 ± 0.02a cm), followed by ‘Papaz Eriği’ (0.60 ± 0.08a cm) and ‘Kayseri Pa’ (0.50 ± 0.04b cm). In addition, the wild plum had the highest number of stamens per flower (23.50 ± 0.50a), slightly exceeding ‘Papaz Eriği’ (22.25 ± 0.96ab) and ‘Kayseri Pa’ (22.00 ± 0.82b) (Supplementary Table S4). Collectively, these findings indicate that the Erciyes wild plum exhibits a partially overlapping floral morphology with both apricot and cultivated plum, while retaining distinct characteristics, particularly a higher stamen number and longer filament length.

**Table 1:** Floral morphological traits (mean ± SD) with Tukey HSD groups

Species	Sepal length (cm)	Sepal width (cm)	Ovary (cm)	Style (cm)	Pedicel length (cm)	Stamen length (cm)	No. of filaments per flower	Petal length (cm)
Plum (Papaz Eriği)	0.60 ± 0.08a	0.40 ± 0.04a	0.30 ± 0.04a	1.00 ± 0.14a	0.35 ± 0.05b	0.60 ± 0.08a	22.25 ± 0.96ab	0.90 ± 0.08b
Wild plum	0.35 ± 0.07b	0.25 ± 0.04b	0.20 ± 0.04b	0.90 ± 0.08a	0.33 ± 0.06b	0.61 ± 0.02a	23.50 ± 0.5a	1.05 ± 0.08ab
Apricot (Kayseri Pa)	0.33 ± 0.03b	0.16 ± 0.05c	0.15 ± 0.04b	0.65 ± 0.13b	0.88 ± 0.10a	0.50 ± 0.04b	22.00 ± 0.82b	1.20 ± 0.18a

*The difference between the means indicated by different letters in the same column is statistically significant (*p* < 0.05)

### Fruit texture

Significant variation (*p* < 0.05) was observed among genotypes for all measured texture parameters (Table 2). The CV, indicating variability among genotypes, was lowest for peel elasticity (PE; 12.53%) and highest for brittleness (B; 48.30%). CV values exceeded 20% for all traits except PE.

Peel hardness (PH) values exceeded 5 N in all wild genotypes, with a mean of 11.59 N, ranging from 5.86 N (#1983) to 21.28 N (#1993). The control cultivar ‘Papaz Eriği’ exhibited values comparable to those of the lowest wild genotypes. The mean PE was 2.50 mm, with the highest value observed in ‘Papaz Eriği’ (3.17 mm) and the lowest in genotype #1986 (1.88 mm).

Genotype #1991 exhibited the highest energy absorption capacity of flesh (EACF; 24.66 mJ), followed by #1988 (22.04 mJ), whereas ‘Papaz Eriği’ showed the lowest value (2.42 mJ). Brittleness (B) values had a mean of 5.02 N mm^−1^, ranging from 2.01 N mm^−1^ (#1983) to 11.86 N mm^−1^ (‘Papaz Eriği’). Resistance of flesh to force (RFF) was highest in genotype #1993 (7.83 mJ), while ‘Papaz Eriği’ recorded one of the lowest values (1.52 mJ). Flesh firmness (FF) ranged from 1.33 N (#1995) to 4.79 N (#1988), with a mean of 2.84 N.

**Table 2:** Texture profile analysis of plum genotypes at harvest time

Genotypes	PH (*N*)	PE (mm)	EACF (mJ)	B (*N*/mm)	RFF (mJ)	FF (*N*)
1779	10.11 ef*	2.83 a–c	14.58 e–h	3.47 g–i	2.80 f–i	2,71 e–i
1971	9.32 e–g	2.63 c	13.46 g–j	3.43 g–i	2.95 e–h	2,38 f–j
1973	6.87 hi	2.71 bc	10.50 i–l	2.39 jk	1.96 hi	2,04 i–k
1974	15.51 c	2.56 c–e	20.30 bc	5.97 d	4.19 b–e	3,76 bc
1975	15.53 c	2.51 c–f	20.68 bc	6.10 cd	4.68 b–d	3,73 b–d
1976	9.97 ef	2.79 bc	14.62 e–h	3.47 g–i	2.81 f–i	2,95 c–h
1977	10.12 ef	2.57 cd	13.75 f–i	3.80 f–h	2.95 e–h	2,62 e–i
1978	12.89 d	2.59 cd	17.19 c–g	4.83 e	3.41 d–g	3,01 c–g
1980	10.94 de	3.01 a	16.25 d–g	3.53 f–h	2.35 g–i	3,18 c–f
1981	12.61 d	2.51 c–f	14.41 e–h	4.78 e	4.00 c–f	2,87 d–i
1982	10.01 ef	2.15 gh	11.73 h–k	4.50 ef	3.50 d–g	2,01 i–k
1983	5.86 i	2.82 a–c	8.57 kl	2.01 k	1.48 i	1,55 jk
1984	10.96 de	2.54 c–e	14.33 e–h	4.24 e–g	3.87 c–f	2,89 c–i
1985	15.16 c	2.12 gh	17.23 c–g	7.03 c	5.16 bc	2,86 d–i
1986	8.68 f–h	1.88 h	8.99 kl	4.39 e–g	4.02 c–f	2,06 h–k
1987	15.41 c	2.21 e–h	17.57 c–e	6.79 cd	5.23 bc	3,41 c–e
1988	15.90 c	2.60 c	22.04 ab	6.28 cd	5.23 bc	4,79 a
1989	6.01 i	2.24 d–g	7.28 l	2.52 i–k	2.22 g–i	1,37 k
1990	8.66 f–h	2.58 cd	11.25 h–k	3.18 h–j	2.72 f–i	2,19 g–k
1991	19.21 b	2.73 bc	24.66 a	6.90 c	5.41 b	4,67 a
1992	15.34 c	2.17 f–h	17.43 c–f	6.80 c	4.77 b–d	2,71 e–i
1993	21.28 a	2.07 gh	19.24 b–d	10.07 b	7.83 a	4,39 ab
1994	6.95 hi	2.18 f–h	8.42 kl	3.12 h–j	5.10 bc	3,29 c–e
1995	7.02 hi	2.63 c	9.88 j–l	2.52 i–k	1.71 hi	1,33 k
Papaz Eriği	7.85 g–i	3.17 a	2.42 m	11.86 a	1.52 i	2.06 h–k
Mean	11.59	2.50	14.26	5.02	3.71	2.84
CV(%)	36.82	12.55	37.19	48.30	41.67	33.85

*The difference between the means indicated by different letters in the same column is statistically significant (*p* < 0.05). PH, peel hardness; PE, peel elasticity; EACF, energy absorption capacity of flesh; B, brittleness; RFF, resistance of flesh to force; FF, flesh firmness; CV, coefficient of variation

### Fruit morphology and total content-based biochemical analysis

Statistically significant differences were observed among plum genotypes in terms of morphological characteristics (Table 3). The CV for the examined traits ranged from 17.64% (fruit length, FL) to 181.35% (a* value). CV values exceeded 20% for fruit weight (FW; 47.52%), flesh thickness (FT; 26.05%), seed weight (SW; 33.09%), L* value (34.25%), a* value (181.35%), and b* value (62.34%), indicating substantial variability among genotypes.

Fruit weight (FW) ranged from 7.33 g (#1988) to 47.53 g (#1971), with a mean value of 22.00 g. Fruit width (Fwidth) varied between 17.67 mm (#1988) and 37.77 mm (#1981), while fruit length (FL) ranged from 18.50 mm (#1988) to 36.37 mm (#1971). Flesh thickness (FT) varied between 4.23 mm (#1988) and 12.53 mm (#1971), and seed weight (SW) ranged from 0.61 g (#1988) to 2.27 g (#1981).

Color parameters also exhibited considerable variation among genotypes. The L* value (lightness) ranged from 20.94 (#1987) to 75.09 (#1989). The a* value, representing the color axis from green (negative values) to red (positive values), varied between − 32.31 (#1976) and 104.17 (#1984). The b* value, indicating the color axis from blue (negative values) to yellow (positive values), ranged from − 0.54 (#1993) to 52.06 (#1990).

**Table 3:** Morphological characteristics analyzed in plum genotypes

Genotype	FW (g)	FWidth (mm)	FL (mm)	FT (mm)	SW (g)	L*	a*	b*
1779	18.62 l*	23.40 f–ı	26.20 de	7.50 ef	1.00 d–h	50.72 c	22.88 c	36.06 c–f
1971	47.53 a	31.30 b	36.37 a	12.53 a	1.93 a	49.90 c	24.84 c	33.76 ef
1973	37.54 c	30.33 bc	31.90 b	10.77 b	1.17 b–f	63.67 b	− 23.75 e–g	43.43 bc
1974	21.95 j	25.83 ef	25.83 d–g	7.43 e–g	1.26 b–e	63.90 b	− 26.28 e–g	42.95 b–d
1975	24.39 h	27.87 c–e	27.77 cd	8.60 c–e	1.35 b–d	63.25 b	− 22.05 e–g	43.36 bc
1976	30.99 e	25.60 e–g	29.60 bc	9.57 bc	1.15 b–f	64.17 b	− 32.31 g	33.77 ef
1977	29.61 f	27.23 de	30.70 bc	9.37 bc	1.26 b–e	51.35 c	19.81 cd	28.19 f
1978	23.18 ı	25.37 e–g	27.73 cd	7.73 d–f	1.16 b–f	67.17 b	− 30.80 fg	46.47 ab
1980	13.88 n	21.93 h–j	20.50 ı–k	5.63 ı–k	0.85 f–ı	52.87 c	7.62 d	35.03 d–f
1981	39.22 b	37.77 a	32.37 b	9.00 cd	2.27 a	32.53 d	80.66 b	11.39 gh
1982	15.46 m	20.87 ıj	23.07 f–ı	6.97 f–ı	0.66 hı	52.92 c	5.88 d	34.82 d–f
1983	28.23 g	29.90 b–d	30.80 bc	8.83 c–e	1.43 b	21.06 f	100.57 a	1.56 ıj
1984	18.52 l	27.90 c–e	25.23 d–g	7.57 d–f	0.82 f–ı	27.31 d–f	104.17 a	8.41 hı
1985	20.26 k	23.87 f–h	26.17 d–f	7.77 d–f	1.16 b–f	47.96 c	31.26 c	18.83 g
1986	13.12 n	20.00 jk	21.27 h–k	5.77 h–j	0.81 f–ı	26.42 d–f	100.81 a	2.59 ıj
1987	10.84 op	20.90 ıj	21.70 h–j	5.00 jk	1.03 c–g	20.94 f	102.00 a	2.19 ıj
1988	7.33 q	17.67 k	18.50 k	4.23 k	0.61 ı	48.32 c	27.10 c	36.50 c–e
1989	21.52 j	22.93 g–ı	24.90 d–g	7.13 f–h	0.95 e–ı	75.09 a	− 13.79 e	30.22 ef
1990	35.10 d	22.07 h–j	24.37 e–h	7.77 d–f	1.11 b–g	66.14 b	− 32.12 g	52.06 a
1991	21.20 jk	25.43 e–g	26.03 d–f	7.67 d–f	1.40 bc	23.48 ef	96.81 a	1.76 ıj
1992	10.97 o	20.90 ıj	22.90 g–ı	6.00 g–j	0.88 f–ı	52.57 c	22.87 c	35.54 c–f
1993	9.80 p	21.30 h–j	19.67 jk	5.03 jk	1.03 d–h	27.52 de	91.74 ab	− 0.54 j
1994	10.82 op	19.37 jk	20.13 ı–k	5.03 jk	0.76 g–ı	54.01 c	17.86 cd	43.37 bc
1995	17.88 l	23.30 f–ı	24.00 e–h	7.03 f–ı	1.12 b–g	62.53 b	− 16.36 ef	51.77 a
Mean	22.00	24.71	25.74	7.50	1.13	48.57	27.48	28.06
CV(%)	47.52	18.49	17.64	26.05	33.09	34.25	181.25	62.34

*The difference between the means indicated by different letters in the same column is statistically significant (*p* < 0.05). FW, fruit weight; FWidth, fruit width; FL, fruit length; FT, flesh thickness; SW, stone weight; CV, coefficient of variation

TPC, TFC, TAC, and AA exhibited considerable variation among plum genotypes (Table 4). TPC ranged from 99.08 (#1973) to 328.30 (#1986), TFC from 5.09 (#1993) to 107.53 (#1975), AA from 22.51% (#1973) to 50.20% (#1986), and TAC from 7.98 (#1973) to 41.94 (#1985). The mean values were 172.63 mg GAE/100 g for TPC, 65.54 mg QE/100 g for TFC, 33.73% for AA, and 18.53 mg cyanidin-3-glucoside/100 g for TAC. The coefficient of variation (CV) indicated substantial variability among genotypes, with values of 78.54% for TAC, 53.81% for TFC, 43.62% for TPC, and 27.78% for AA.

**Table 4:** Biochemical characteristics analyzed in plum genotypes

Genotype	TPC (mg GAE/100 g)	TFC (mg QE/100 g)	AA (% DPPH)	TAC (mg cyn-3-gluc/100 g)
1779	102,80 lm*	92,85 cd	25,93 k	8,38 fg
1971	103,64 l	93,36 c	25,59 k	8,08 fg
1973	99,08 n	80,80 f	22,51 l	7,98 g
1974	101,10 l-n	81,82 f	23,43 l	8,52 e-g
1975	168,11 ı	107,53 a	33,76 ı	9,67 e
1976	101,25 l-n	82,16 f	23,41 l	8,36 fg
1977	114,29 k	91,84 cd	39,66 g	8,40 fg
1978	100,70 mn	81,82 f	23,62 l	8,31 fg
1980	101,68 l-n	91,65 d	26,60 k	8,82 e-g
1981	255,39 d	12,53 j	42,82 de	40,87 a
1982	190,08 h	85,39 e	34,44 ı	8,79 e-g
1983	230,40 e	21,13 h	40,97 fg	36,67 d
1984	271,45 c	22,43 h	45,48 b	38,42 bc
1985	253,24 d	13,46 j	43,76 cd	41,94 a
1986	328,30 a	30,34 g	50,20 a	37,89 c
1987	271,44 c	22,45 h	45,56 b	38,32 bc
1988	195,47 g	85,55 e	36,98 h	8,16 fg
1989	100,13 mn	81,87 f	23,11 l	8,13 fg
1990	99,91 n	81,30 f	22,57 l	8,13 fg
1991	214,31 f	18,05 ı	42,10 ef	35,69 d
1992	163,73 j	108,07 a	31,30 j	8,79 e-g
1993	282,40 b	5,09 k	45,01 bc	39,13 b
1994	194,65 g	99,92 b	37,54 h	9,25 ef
1995	99,54 n	81,55 f	23,06 l	8,08 fg
Mean	172,63	65,54	33,73	18,53
CV(%)	43.62	53.81	27.78	78.54

*The difference between the means indicated by different letters in the same column is statistically significant (*p* < 0.05). TPC, total phenolic content; TFC, total flavonoid content; AA, antioxidant activity; TAC, total anthocyanin content; CV, coefficient of variation

### Relationships between genotypes and examined traits

**Fig. 2:**
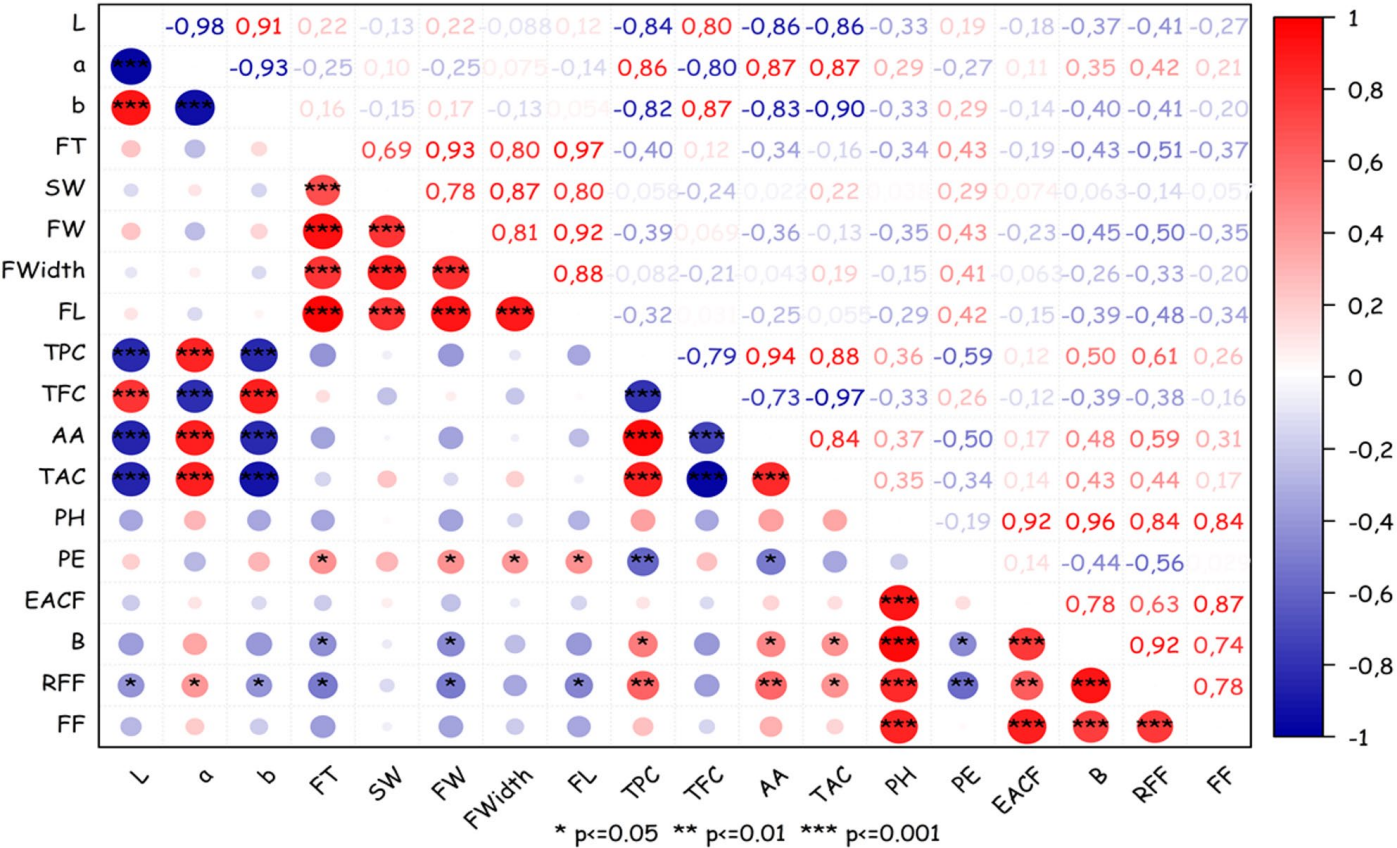
Pearson correlation matrix of morphological, TPC, TFC, TAC, and AA, color, and texture traits in plum genotypes. Only strong correlations (*p* < 0.01) are presented to reduce the risk of Type I error associated with multiple testing

Statistically significant positive and negative correlations were identified among fruit traits, with only strong relationships presented in Fig. 2. A strong positive correlation was observed between L* and b* (*r* = 0.91) as well as between L* and TFC (*r* = 0.80), whereas L* was negatively correlated with a* (*r* = − 0.98). Similarly, a* exhibited a strong negative correlation with b* (*r* = − 0.93). Significant positive correlations were detected among fruit dimensional traits. The strongest associations were observed between FT and FL (*r* = 0.97), FT and FW (*r* = 0.93), and FW and FL (*r* = 0.92). TPC, TFC, TAC, and AA parameters also showed significant interrelationships. TPC was positively correlated with AA (*r* = 0.94) and TAC (*r* = 0.88), while AA and TAC were also positively associated (*r* = 0.84). In contrast, TPC showed a negative correlation with TFC (*r* = − 0.79), and TFC was negatively correlated with AA (*r* = − 0.73) and TAC (*r* = − 0.97). Significant correlations were also observed between TPC, TFC, TAC, and AA and color parameters. The a* value was positively correlated with TPC (*r* = 0.86), AA (*r* = 0.87), and TAC (*r* = 0.87), whereas it was negatively correlated with TFC (*r* = − 0.80). In contrast, L* and b* values exhibited predominantly negative correlations with TPC, TFC, TAC, and AA, except for TFC. Regarding texture profile traits, generally strong positive correlations were observed. The highest positive correlations were detected between PH and B (*r* = 0.96), PH and EACF (*r* = 0.92), and B and RFF (*r* = 0.92). Among these traits, negative correlations were observed between PE and RFF (*r* = − 0.56) and between PE and B (*r* = − 0.44).

**Fig. 3:**
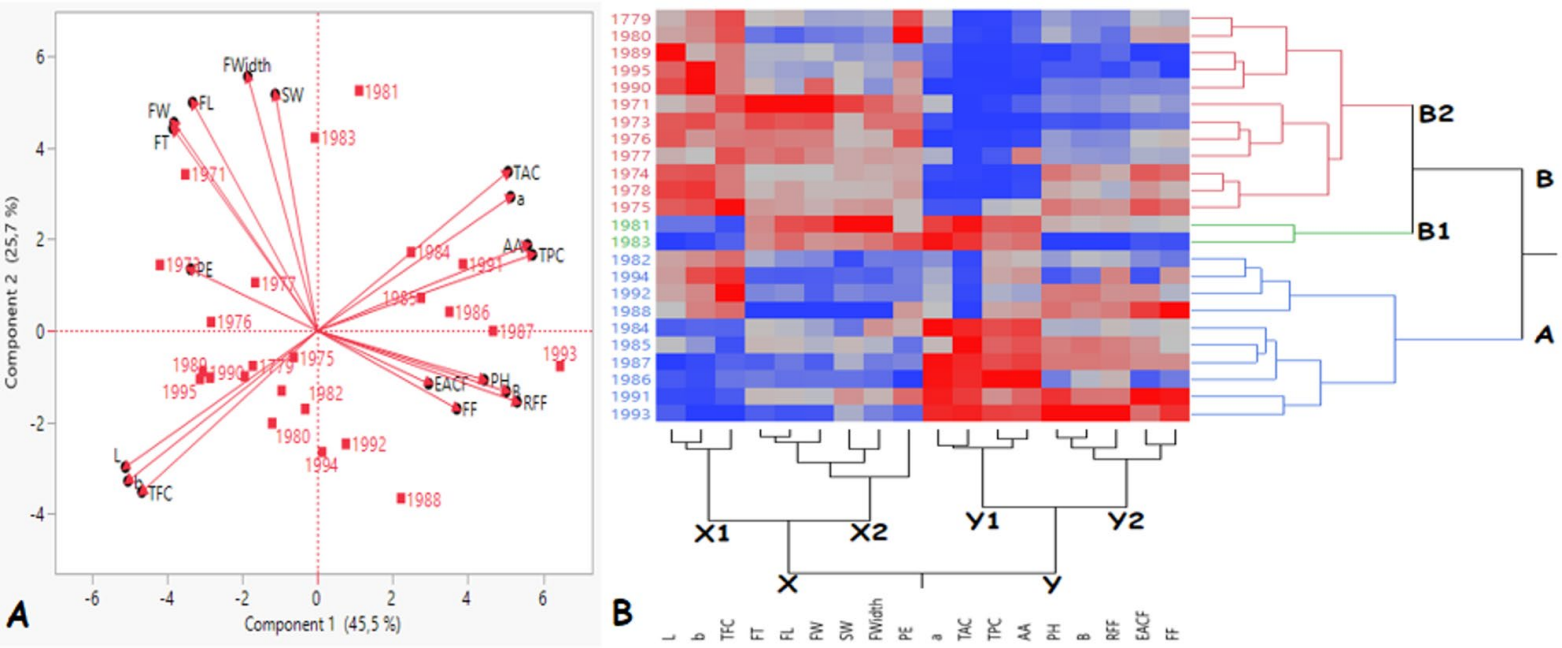
PCA **A** and heatmap hierarchical clustering analysis **B** graphs of plum genotypes and examined traits

Heatmap hierarchical clustering and principal component analysis (PCA) are widely used statistical approaches for evaluating trait variation and interpreting complex datasets. According to the PCA results, the first two principal components (PC1 and PC2), which explain a substantial proportion of the variation among genotypes, accounted for 71.2% of the total variance. Hierarchical clustering analysis based on the heatmap grouped the genotypes into two main clusters according to the examined traits. Each main cluster was further subdivided into two subclusters. Genotypes #1982, #1984, #1985, #1986, #1987, #1988, #1991, #1992, #1993, and #1994 formed Group A, whereas the remaining genotypes were assigned to Group B. Within Group B, genotypes #1981 and #1983 were distinctly separated from the others, forming a subcluster (B1). In the heatmap, the color gradient from blue to red indicates increasing trait values. The examined traits were also grouped into two main clusters, each comprising two subgroups. Genotypes in Group A were characterized by higher values for a*, TAC, TPC, AA, PH, B, RFF, EACF, and FF (Group Y traits). In contrast, genotypes in subcluster B2 exhibited higher values for L*, b*, TFC, FT, FL, FW, SW, FWidth, and PE (Group X traits). Genotypes in subcluster B1 showed relatively high values for FT, FL, FW, SW, FWidth, PE, a*, TAC, TPC, and AA, corresponding to the X2 and Y1 trait groups (Fig. 3).

### Chloroplast genome characterization

**Fig. 4:**
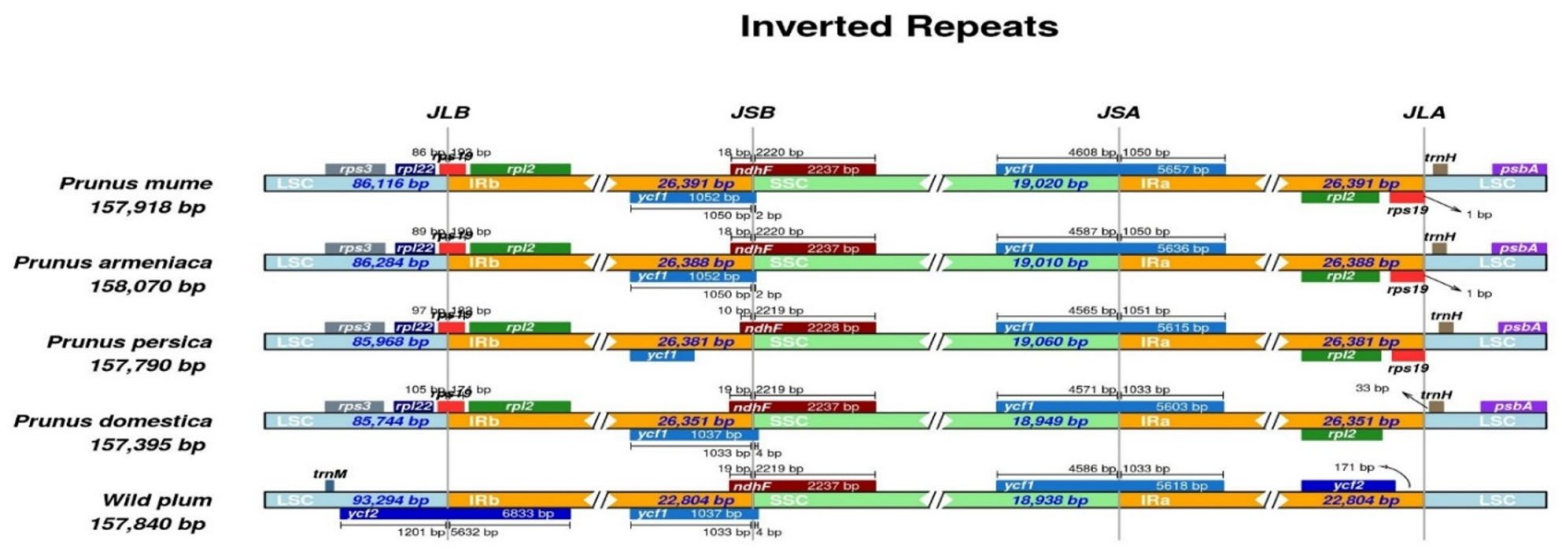
Comparative analysis of inverted repeat (IR) regions in the plastome of Erciyes wild plum and selected *Prunus* species. Gene distribution and region lengths within the IRa, IRb, SSC, and LSC regions are illustrated. JSB indicates the junction between IRb and SSC, JLB marks the boundary between LSC and IRb, JSA represents the transition between SSC and IRa, and JLA denotes the junction between IRa and LSC

High-throughput paired-end sequencing using the Illumina NovaSeq 6000 platform generated more than 150 million raw reads across the two wild plum accessions. The red-fruited accession (GenBank accession number: SRR35609914) yielded 92.5 million reads, corresponding to 27.7 Gb of sequence data, whereas the yellow-fruited accession (SRR35609915) produced 43.8 million reads totaling 13.1 Gb. The overall GC content of the raw data was approximately 37%. Sequencing quality was high, with more than 95.7% of bases exceeding Q20 and 91.2% exceeding Q30. Chloroplast genome assembly achieved high and uniform coverage for both accessions. The red-fruited accession exhibited a mean plastome coverage depth of 1265×, with a minimum coverage of 96×, whereas the yellow-fruited accession showed a mean coverage depth of 577× and a minimum coverage of 32×. No assembly gaps were detected in either plastome, indicating the completeness and reliability of the assembled chloroplast genomes. The nucleotide sequences of the plastomes assembled from the red- and yellow-fruited accessions were identical. The plastome of the sampled individuals exhibited the typical quadripartite structure, with a total length of 157,840 bp, comprising a large single-copy (LSC) region of 93,294 bp, a small single-copy (SSC) region of 18,938 bp, and a pair of inverted repeats (IRs) of 22,804 bp each (Supplementary Table S6). The overall GC content was 36.8%. A total of 125 genes were annotated, including 81 protein-coding genes, 36 tRNA genes, and 8 rRNA genes (Supplementary Table S7). Gene order and overall structure were largely conserved compared with other *Prunus* plastomes; however, minor structural differences were observed. The LSC region contained *trnM* and *ycf2*, while the SSC region contained *ycf2* instead of *rpl2* and *psbA*. In contrast, the IR regions remained highly conserved (Fig. 4; Supplementary Fig. S1).

### Phylogenetic analysis

**Fig. 5:**
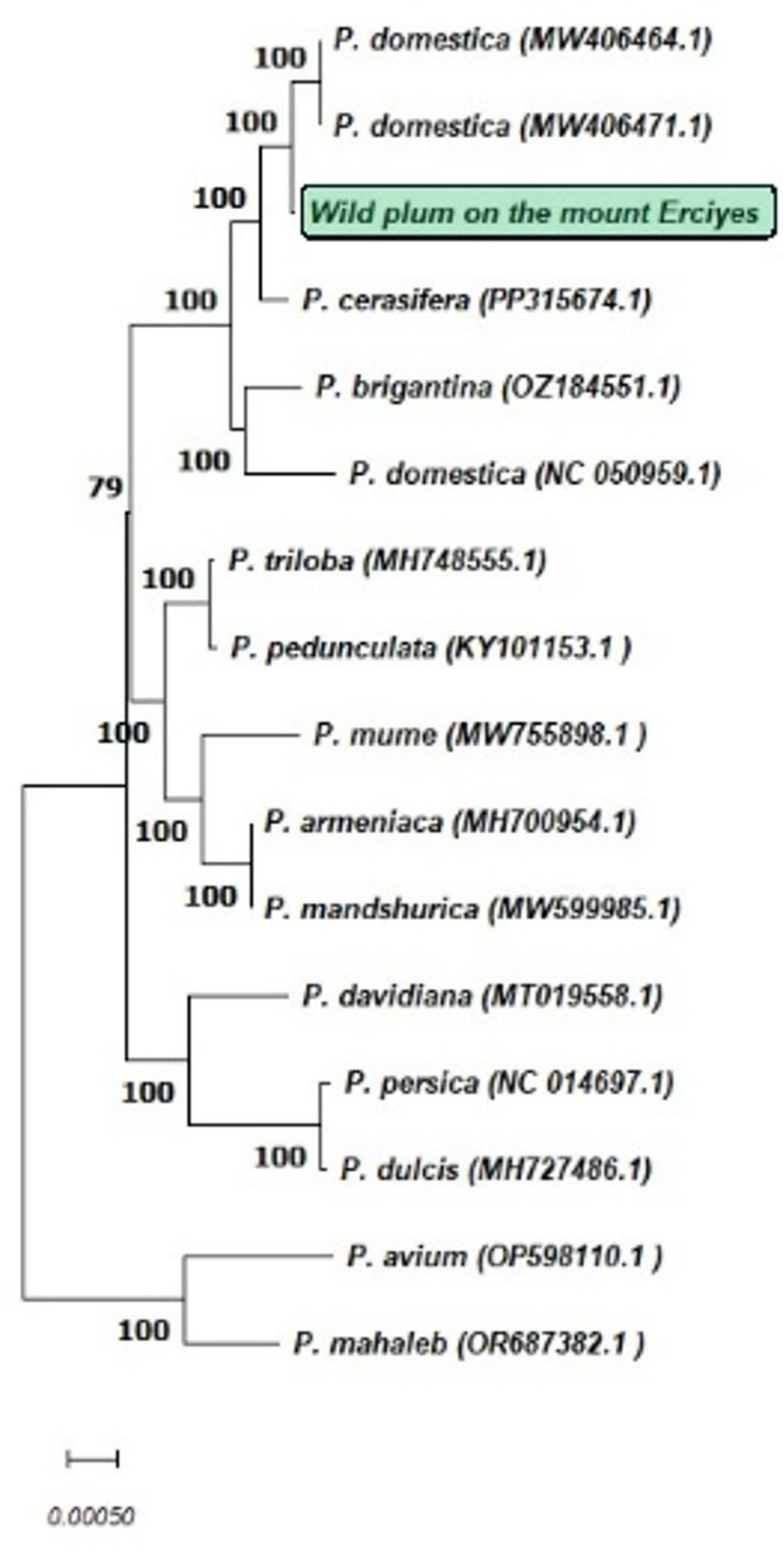
Maximum-likelihood phylogenetic tree of *Prunus* spp. inferred from complete plastome sequences. Node support was evaluated using 1,000 bootstrap replicates in MEGA12. The Erciyes wild plum is highlighted in green

The maximum-likelihood phylogenetic tree constructed using complete plastome sequences resolved the major *Prunus* lineages with strong bootstrap support. The sampled individualsclustered with *P. domestica* and *P. cerasifera*, forming a well-supported clade distinct from *P. salicina* and other East Asian taxa. High bootstrap support (100%) confirmed its placement within the European plum lineage, indicating a strong plastome affinity with the maternal lineage of cultivated plums (Fig. 5).

## Discussion

This study was designed as an integrative characterization rather than a hypothesis-driven investigation focusing on a single trait category. Plastome analysis provides a phylogenetic framework upon which morphological, textural, and TPC, TFC, TAC, and AA were measured to describe the Erciyes wild plum. Although each analytical layer addresses a distinct aspect of plant biology, their combined evaluation enables a more comprehensive understanding of the wild plums than any single approach alone.

Floral analyses revealed that the Erciyes wild plum exhibits overlapping characteristics with both apricot and cultivated plum, including white petals with lengths comparable to those of both species and sepal dimensions more closely resembling those of plum. Similar patterns have been reported for apricot and plum floral traits [39, 40], suggesting that wild plum may represent transitional morphotypes. The relatively lower stamen number compared to apricot, but similar to that of cultivated plum, further supports its intermediate floral position, consistent with observations in other *Prunus* germplasm [41]. However, the observed overlap in floral traits may also reflect shared developmental constraints within *Prunus*, rather than direct evolutionary intermediacy.

Fruit texture analysis revealed substantial variability among wild genotypes, with several individuals exhibiting higher PH and RFF than the commercial cultivar ‘Papaz Eriği’. These traits are important for postharvest quality and consumer acceptance, as PH is negatively associated with PE but positively correlated with B, EACF, and FF. These patterns are consistent with previous reports on texture variation in plums and other fruit crops [42, 43]. The superior texture characteristics observed in certain wild genotypes suggest that Erciyes plums may serve as valuable genetic resources for improving firmness and storage potential in cultivated plums. In addition, higher PH values in wild genotypes may be associated with delayed softening and adaptation to abiotic stress conditions, rather than traits selected through domestication.

The CV ranged from 12.55% (PE) to 181.25% (a*) in the present study, with most traits exhibiting CV values greater than 20%. Traits with CV values exceeding 20% indicate substantial variability among genotypes and are therefore useful for genotype discrimination. In contrast, traits with lower CV values are more stable across genotypes. High CV values also suggest a broader selection potential for breeding programs, whereas low CV values indicate trait stability [44, 45]. Previous studies on plum genotype characterization have reported CV ranges between 7.43% and 81.65% [46], 8.18% and 45.39% [47], 16.33% and 194.93% [48], and 5.23% and 109.40% [49]. The high variability observed across most morphological traits and in TPC, TFC, and TAC, as reflected by elevated CV values, suggests a heterogeneous population structure with substantial phenotypic plasticity. Rather than simply indicating variation, this pattern points to the presence of genotypes with distinct adaptive and functional traits, which may be shaped by both genetic background and microenvironmental conditions. From a breeding perspective, such variability represents an important resource, as traits with wider dispersion provide greater opportunities for selection and improvement. Moreover, the magnitude of variation observed in the present study is comparable to or exceeds that reported in previous studies on plum germplasm [46–49], indicating that the Erciyes plum population constitutes a particularly diverse and potentially valuable genetic pool.

Fruit traits provide essential information for the characterization of plum cultivars and genotypes [50]. Among these traits, FW and SW are primary breeding criteria due to their economic importance. FW is a key pomological trait influenced by multiple factors, including genetic background, nutritional status, crop load, and bud position on the branches [51]. In previous studies on plum genotypes, FW and SW were reported as 1.79–14.09 g and 0.25–1.01 g by Khadivi et al. [52], 4.97–42.19 g and 0.37–1.70 g by Mirheidari et al. [51], 12.68–56.9 g and 0.71–2.45 g by Borji and Rezaei [47], 6.63–25.92 g and 0.37–1.05 g by Sümbül et al. [53], and 5.56–29.79 g and 0.31–1.21 g by Say et al. [48].

Fruit morphological traits are also important determinants of consumer preference [54]. Consumers generally prefer plum fruits with a round shape and without pronounced protrusions at the ends [55]. Dimensional traits such as FWidth, FL, and FT are closely associated with FW and SW. In the present study, plum genotypes predominantly exhibited round-shaped fruits consistent with consumer preferences. Similar findings have been reported in previous studies [46, 48, 52].

Fruit peel color is one of the most critical attributes influencing fruit quality and consumer appeal. However, peel color is affected by multiple factors, including tree position, climatic conditions, growth habit, light distribution, and genetic background [56]. Environmental conditions of the growing region play a particularly important role in color development [51]. Peel color is also commonly used as an indicator of fruit maturity [46]. The plum genotypes examined in this study exhibited considerable variation in peel color. It is known that fruit peel color is controlled by a limited number of genes, with dark coloration generally dominant over lighter tones [46]. Consistent with our findings, previous studies have reported a wide range of fruit colors in plum genotypes, from yellowish-green to red and even black [52, 53, 57].

Statistically significant differences were detected among plum genotypes for TPC, TFC, AA, and TAC. Previous studies have reported substantial variation in TPC, TFC, TAC, and AA, among plum cultivars and genotypes. Trendafilova et al. [58] reported TPC values ranging from 93.7 to 156.1 mg GAE/100 g, TFC from 11.80 to 16.60 mg QE/100 g, AA (DPPH) from 6.49 to 9.40 mg/mL, and TAC from 9.60 to 19.20 mg cyanidin-3-glucoside/100 g. Taşkın and Ercisli [49] reported TPC values between 25.76 and 451.14 mg GAE/100 g, AA (DPPH) between 5.13% and 81.02%, TFC between 1.68 and 4.89 mg QUE/g, and TAC between 0.11 and 1.01 mg cyanidin-3-glucoside/100 g. Similarly, Sümbül et al. [53] reported TPC values ranging from 99.98 to 327.01 mg GAE/100 g, TFC from 5.17 to 109.98 mg QE/100 g, AA from 23.00% to 48.70%, and TAC from 7.99 to 44.54 mg cyanidin-3-glucoside/100 g.

There is increasing interest in the antioxidant activity of fruits, largely attributed to phenolic and flavonoid compounds [59]. Previous studies have demonstrated that fruit peels are richer in bioactive compounds, including TPC, TFC, and TAC [58, 60–62]. Therefore, consumption of fruits together with their peel may provide additional health benefits [45]. Antioxidants play a crucial role in neutralizing free radicals and may contribute to the prevention of various diseases, including cancer and cardiovascular disorders [63, 64]. The wide variation observed among genotypes in TPC, TFC, TAC, and AA likely reflects both genetic diversity and microenvironmental heterogeneity within the wild plums, as reported for other wild *Prunus* species. Accordingly, plum genetic resources represent valuable materials for breeding programs due to their health-promoting properties, functional food potential, and distinctive phenotypic traits.

The ecological setting of Mount Erciyes may also help explain part of the observed phenotypic variation and differences in TPC, TFC, TAC, and AA, in this wild plums. The sampled trees grow in a mountainous habitat where high elevation, seasonal temperature fluctuations, relatively dry conditions, and heterogeneous light exposure may impose environmental pressures different from those experienced by cultivated plum orchards. Such environmental conditions are known to influence fruit texture and the accumulation of secondary metabolites through stress-induced physiological responses [65]. Under these conditions, increased peel hardness and flesh resistance may represent adaptive traits associated with delayed softening, protection against mechanical damage, and reduced water loss. Similarly, the wide variation in total phenolic content, antioxidant activity, and anthocyanin accumulation may reflect both genetic differentiation among wild genotypes and environmentally induced responses to abiotic stress, including high irradiance, temperature variation, and drought-related stress, which have been shown to enhance phenolic and anthocyanin biosynthesis in fruit crops [66, 67]. The association between red fruit pigmentation, higher a* values, and increased phenolic and anthocyanin-related indices further suggests that fruit color differentiation may be linked to these compounds, which are known to play roles in stress protection and ecological function. However, because detailed microclimatic and soil data were not collected for each individual tree, these ecological interpretations should be regarded as hypotheses. Future studies combining fine-scale environmental measurements, replicated population sampling, and nuclear genomic data will be necessary to test whether the observed trait variation reflects local adaptation, phenotypic plasticity, or both.

Understanding the relationships among the examined traits is essential for breeding programs. In this study, significant positive and negative correlations were identified among morphological traits, TPC, TFC, TAC, and AA. The correlation patterns observed were consistent with previous findings in plum studies [46, 48, 49, 51–53, 58, 60, 64].

While heatmap analysis evaluates all examined traits and genotypes collectively, PCA assesses the relative contribution of the examined traits within the scope of the study. These approaches facilitate the evaluation of germplasm in plant species. PCA identifies the key traits driving differentiation among genotypes, enabling the assessment of population structure and the distribution of individuals within the population. The proportion of variance explained by the first two principal components (71.20%) in the present study exceeds those reported by [46], Mirheidari et al. [51], Khadivi et al. [52], Sümbül et al. [53], Say et al. [48], and Taşkın and Ercisli [49]. The separation of genotypes into two main groups (Group A and Group B) revealed by hierarchical clustering appears to be biologically meaningful and associated with distinct trait combinations. Genotypes in Group A were generally characterized by higher a* together with elevated TPC, TAC, and AA, suggesting a relationship between red fruit pigmentation and increased accumulation of phenolic and anthocyanin-related compounds. In contrast, Group B genotypes exhibited higher L* and b*, indicating lighter and more yellow fruit coloration, and were also associated with relatively higher TFC and fruit size-related traits. These patterns suggest that the observed clustering may reflect coordinated variation in fruit color, TPC, TFC, TAC, and AA, and morphological traits.

Chloroplast genome sequencing revealed that the Erciyes wild plum plastome retains the conserved quadripartite structure typical of *Prunus*, comprising 125 genes and a GC content of 36.8%. Gene content was largely consistent with previously published plastomes of *P. persica*, *P. mume*, and *P. tomentosa* [68, 69], although minor structural variations were observed in the LSC and SSC regions. Chloroplast genomes are generally highly conserved in angiosperms due to their uniparental inheritance, low mutation rates, and limited recombination [68, 69]. Accordingly, plastome sequences often show minimal variation among individuals within local populations of woody perennials, including *Prunus* [15, 70], limiting their resolution for detecting intra-population diversity. These differences primarily involved shifts in gene boundary positions rather than gene loss or large-scale rearrangements. Genes such as *ycf2*, *rpl2*, and *psbA* are typically located near IR–LSC or IR–SSC junctions and frequently exhibit positional variation among closely related *Prunus* plastomes. Such boundary shifts are widely attributed to expansion–contraction dynamics of IR regions, a well-documented feature of plastome evolution in angiosperms rather than evidence of adaptive or lineage-specific divergence [68–69]. Accordingly, these variations are interpreted as structural polymorphisms, while the highly conserved IR regions further support their stabilizing role in plastome evolution.

Phylogenetic analysis placed the Erciyes wild plum within a strongly supported clade together with *P. domestica* and *P. cerasifera*, and distinct from East Asian taxa such as *P. salicina*. This finding is consistent with previous evidence indicating that *P. cerasifera* is the maternal progenitor of European plum [3, 15]. Rather than implying direct ancestry or domestication pathways, the observed plastome affinity positions the Erciyes wild plum within the broader European plum lineage and provides a phylogenetic context for its distinct morphological traits and associated variation in TPC, TFC, TAC, and AA. From a broader evolutionary perspective, this plastome affinity may reflect either retention of ancestral maternal lineages or historical gene flow between wild and cultivated *Prunus* species, both of which have been frequently reported in perennial fruit crops. In this context, the Erciyes plum population may contribute to maintaining genetic diversity within the European plum lineage and may represent important reservoirs of adaptive traits relevant for breeding and conservation. Although Anatolia is geographically close to regions historically proposed as centers of plum domestication [4], the maternally inherited nature of plastid genomes limits the ability of plastome data alone to resolve domestication origins or ancestral relationships. Therefore, the present results should be interpreted as evidence of phylogenetic proximity rather than proof of progenitor status. A comprehensive understanding of plum domestication will require extensive sampling across wild and cultivated populations, combined with nuclear genomic data and archaeobotanical evidence. Within this framework, the Erciyes wild plum represents a well-defined wild lineage that may serve as a valuable reference for future evolutionary, conservation, and breeding studies.

In conclusion, this study provides a comprehensive characterization of a previously undocumented wild plum from Mount Erciyes by integrating floral, textural, total content-based biochemical profiling, and plastome-level phylogenetic analyses. The combined phenotypic and plastome evidence highlights the distinctiveness of this wild plum while situating it within the broader European plum lineage. Importantly, plastome data are used here to establish phylogenetic context rather than to infer population structure or domestication history.

By documenting substantial variation in morphological traits and TPC, TFC, TAC, and AA, within this wild plum, the present study establishes a baseline reference for future research on plum evolution, conservation, and breeding. The Erciyes wild plum thus represents a valuable wild genetic resource, and its detailed characterization may facilitate subsequent studies incorporating broader sampling and nuclear genomic data to address questions beyond the scope of the present work.

## Conclusion

This study provides a comprehensive and integrative characterization of a previously undocumented wild plum (*Prunus* spp.) from Mount Erciyes (Central Anatolia, Türkiye) by combining morphological, textural, TPC, TFC, TAC, and AA, and plastome-level analyses. The results demonstrate that this wild plum exhibits substantial phenotypic and TPC, TFC, TAC, and AA variability, highlighting its distinctiveness within the broader European plum lineage.

Plastome-based phylogenetic analysis positioned the Erciyes wild plums in close association with *P. domestica* and *P. cerasifera*, confirming their affinity with the European plum maternal lineage while providing a robust evolutionary framework for interpreting observed trait variation. Importantly, plastome data were used to establish phylogenetic context rather than to infer domestication pathways.

The significant variability detected in fruit texture, morphological attributes, and bioactive compound composition indicates a high potential for the utilization of this wild germplasm in breeding programs. In particular, traits related to fruit firmness, antioxidant capacity, and phenolic content suggest that these genotypes may serve as valuable genetic resources for improving fruit quality, postharvest performance, and nutritional value in cultivated plums.

Overall, the Erciyes wild plum population represents a valuable and underexplored genetic resource. Its detailed characterization not only contributes to the understanding of plum evolutionary diversity but also establishes a baseline for future studies integrating broader sampling strategies and nuclear genomic approaches. Such efforts will be essential for elucidating domestication processes, enhancing conservation strategies, and supporting the development of climate-resilient and high-quality plum cultivars.

## Supplementary Information

Below is the link to the electronic supplementary material.

Supplementary Material 1

## Data Availability

The plastome data of the de novo assembled wild plum generated and analyzed during the current study were submitted to GenBank (accession number: PX421575). The raw sequencing reads of two wild plums generated in this study have been deposited in the GenBank repository under BioProject: PRJNA1332929.

## Declarations

### Statement specifying permissions

For this study, we acquired permission to study plum issued by the Agricultural and Forestry Ministry of the Republic of Türkiye.

### Statement on experimental research and field studies on plants

The either cultivated or wild-growing plants sampled comply with relevant institutional, national, and international guidelines and domestic legislation of Türkiye.
