# Supplementary material for: Integrative morphological, biochemical, and plastome-based characterization of a wild plum (Prunus spp.) population from Mount Erciyes (Central Anatolia, Türkiye)
Source: Biol Res. 2026 May 27;59:42. doi: 10.1186/s40659-026-00702-0 (PMC13404573; doi:10.1186/s40659-026-00702-0)
Supplement: Supplementary file 1 — Supplementary Material 1 [file 40659_2026_702_MOESM1_ESM.doc]

**Supplementary Table S1.** Comparative analysis of sepal morphology in the local apricot cultivar ‘Kayseri Pa’, *Prunus* spp., and the control plum ‘Papaz Eriği’.

| Apricot | *Prunus* spp. | Plum |
| --- | --- | --- |
| 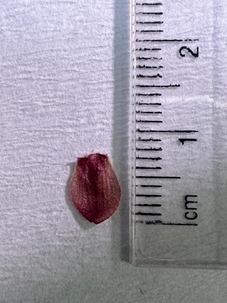  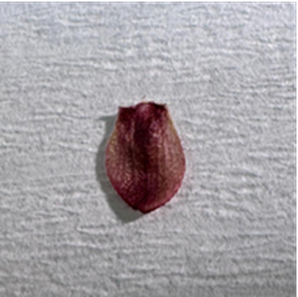 | 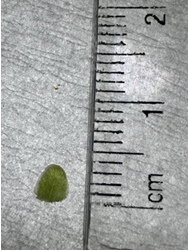  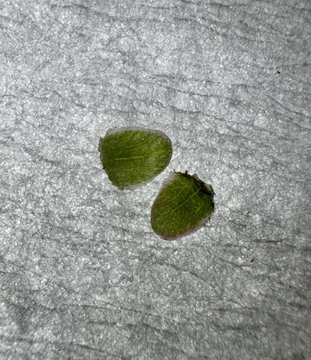 | **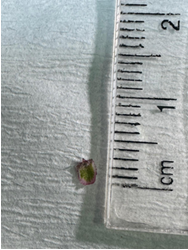**  **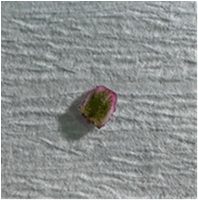** |
| Sepal color | | |
| Maroon | Green | Pale green |
| Sepal length (cm | | |
| 0.32 ± 0.03 c | 0.60 ± 0.08 a | 0.35 ± 0.07 b |
| Sepal width (cm) | | |
| 0.16 ± 0.05 c | 0.40 ± 0.04 a | 0.35 ± 0.07 b |

This photograph was captured as part of the present study and was provided by the second author, Prof. Dr. Kahraman Gürcan. It is not subject to any third-party copyright restrictions.

**Supplementary Table S2.** Morphological characteristics of the stigma and ovary in the local apricot cultivar ‘Kayseri Pa’, *Prunus* spp., and the control plum ‘Papaz Eriği’.

| Apricot | *Prunus* spp. | Plum |
| --- | --- | --- |
| **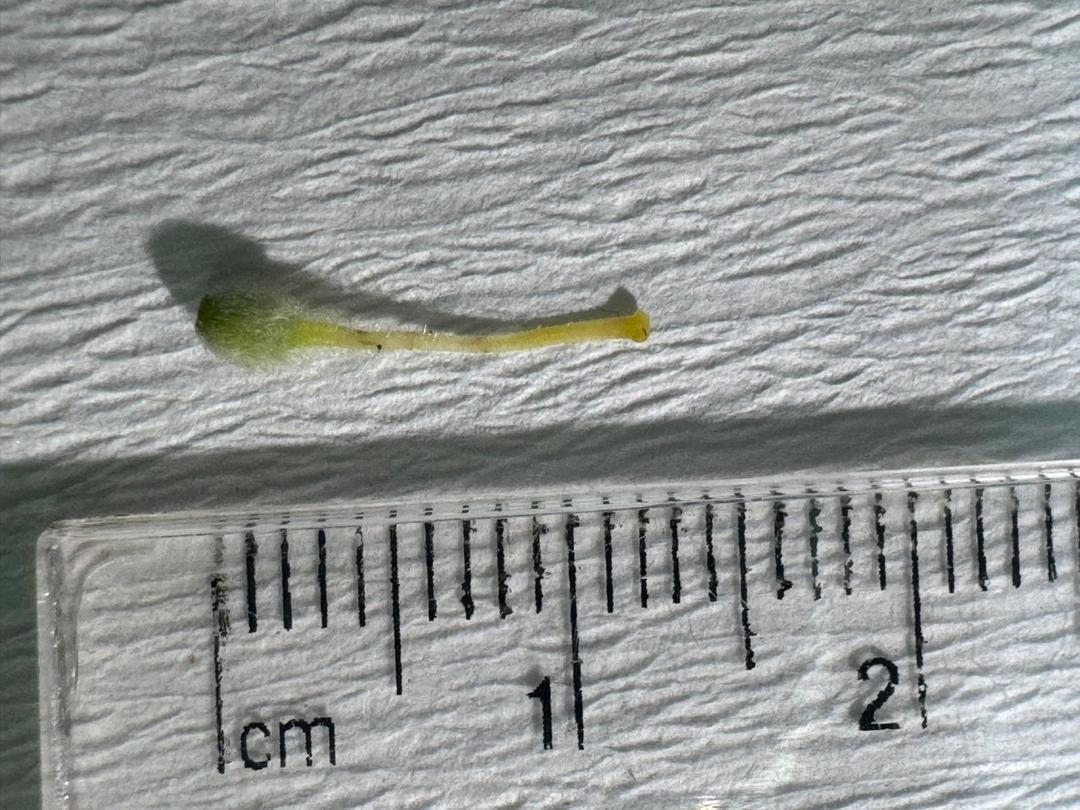**  **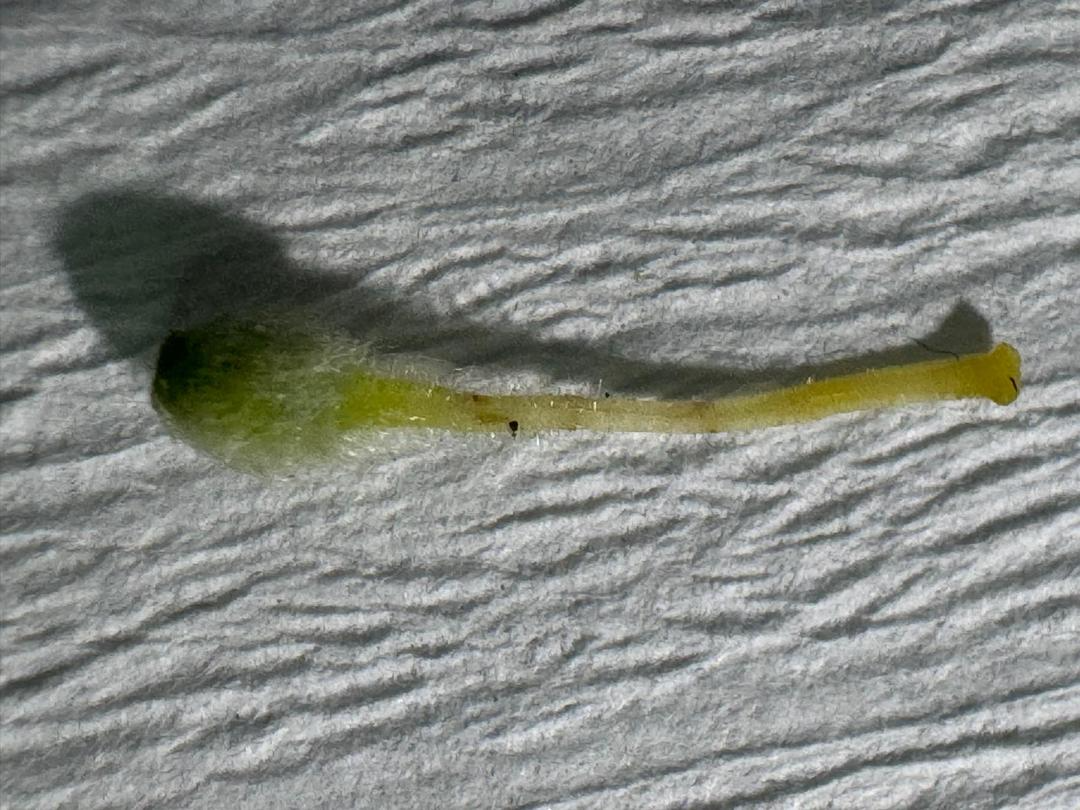** | **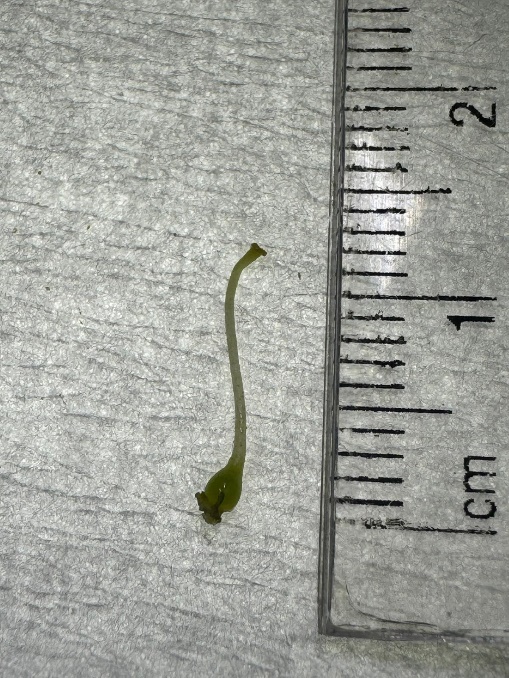**  **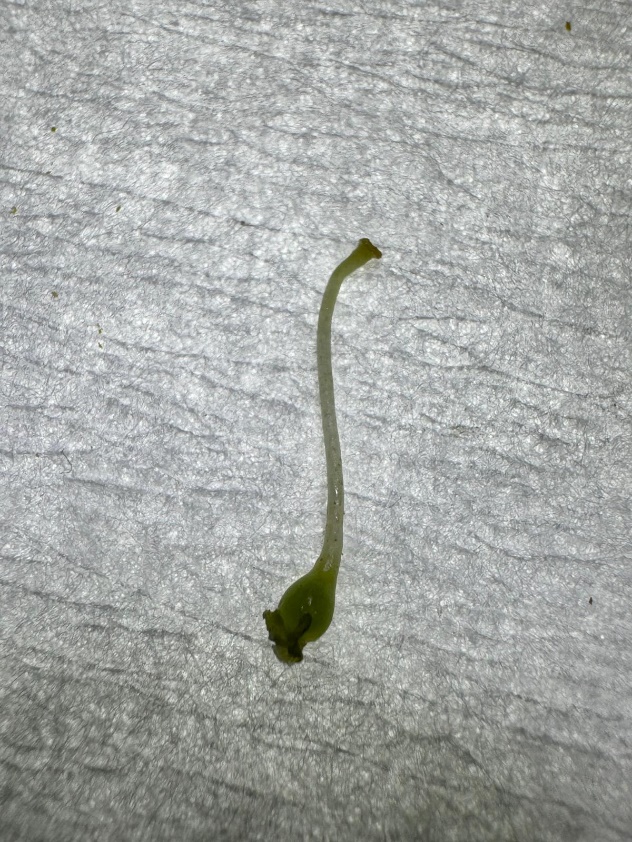** | **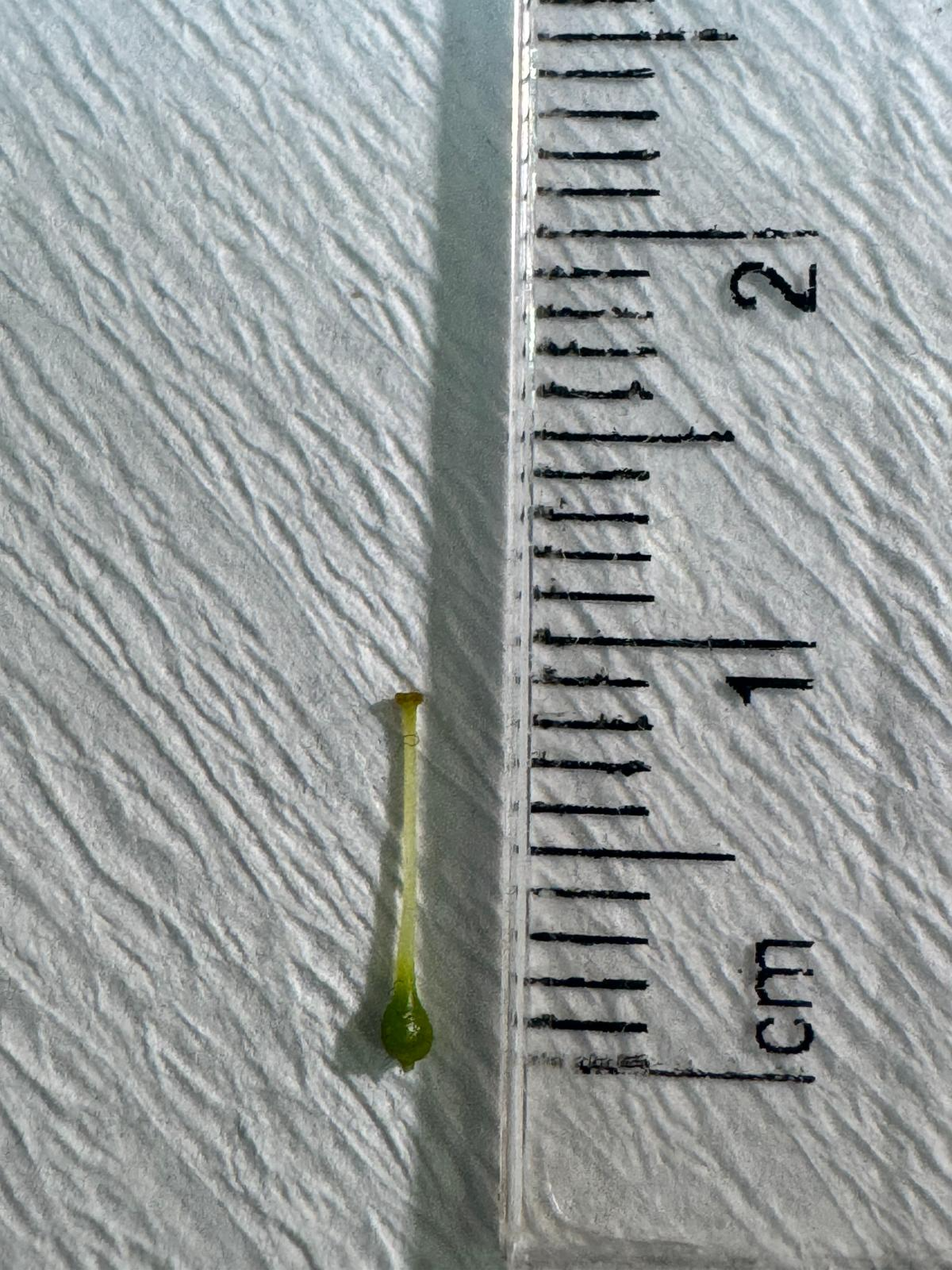**  **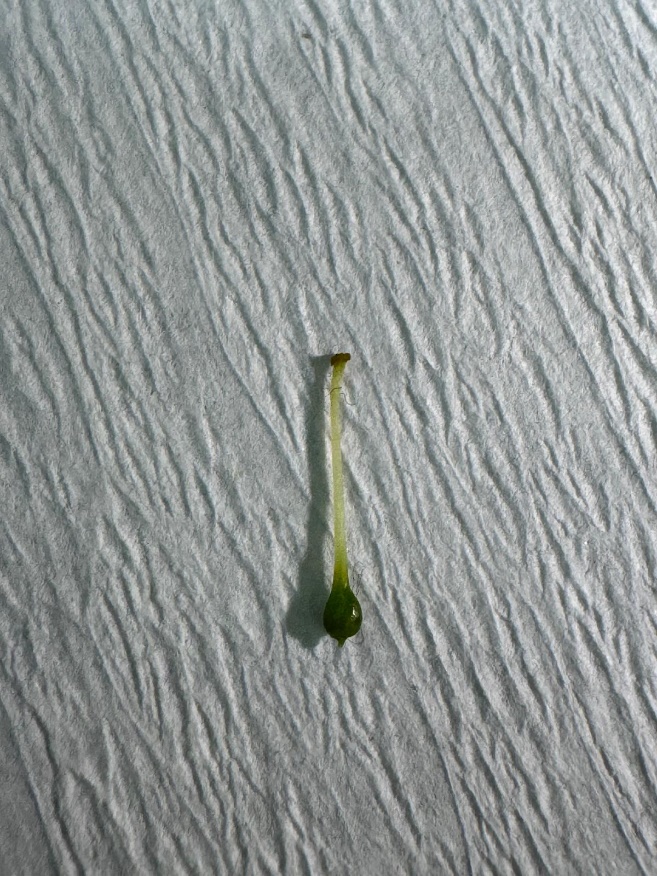** |
| Ovary characteristics | | |
| Large, hairy ovary | Medium, smooth ovary | Small, smooth ovary |
| Style characteristics | | |
| Long, thick style | Medium, smooth style | Long, thin style |
| Stigma characteristics | | |
| Large, sticky stigma | Medium, sticky stigma | Small, sticky stigma |
| Ovary length (cm) | | |
| 0.15 ± 0.04 c | 0.20 ± 0.04 b | 0.30 ± 0.04 a |
| Style length (cm) | | |
| 0.65 ± 0.13 c | 0.90 ± 0.08 b | 1.00 ± 0.14 a |

This photograph was captured as part of the present study and was provided by the second author, Prof. Dr. Kahraman Gürcan. It is not subject to any third-party copyright restrictions.

**Supplementary Table S3.** Morphological characteristics of the pedicel in the local apricot cultivar ‘Kayseri Pa’, *Prunus* spp., and the control plum ‘Papaz Eriği’.

| Apricot | *Prunus* spp. | Plum |
| --- | --- | --- |
| **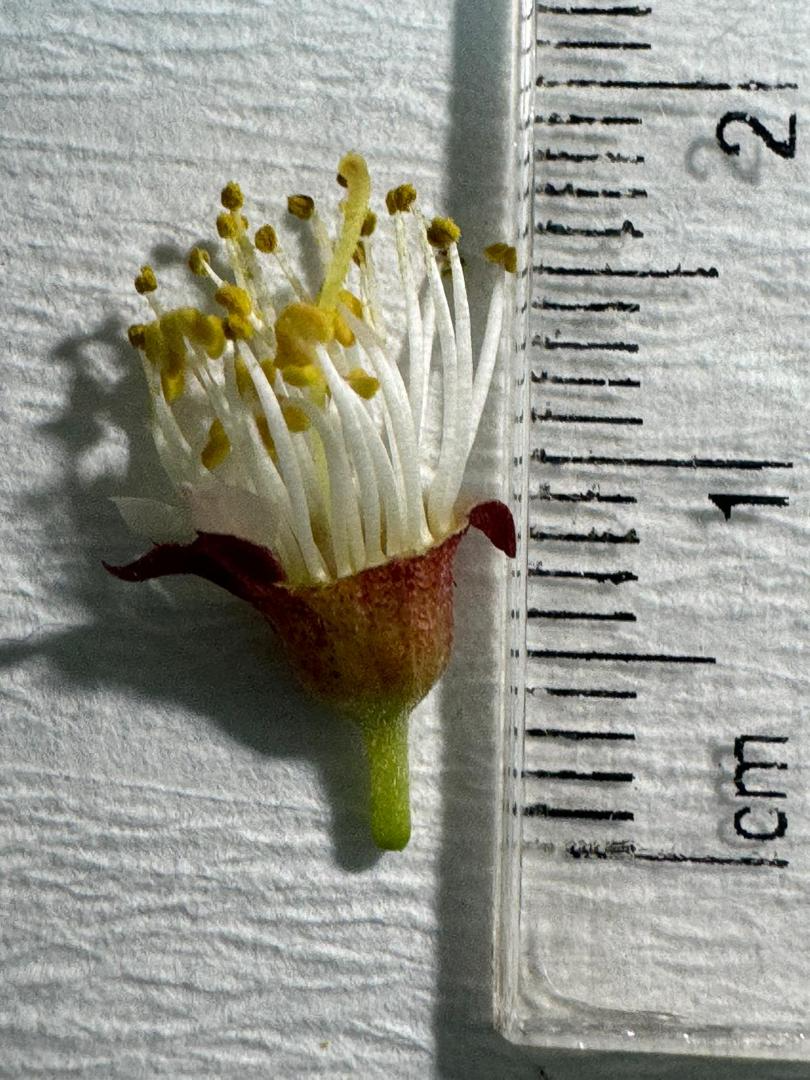**  **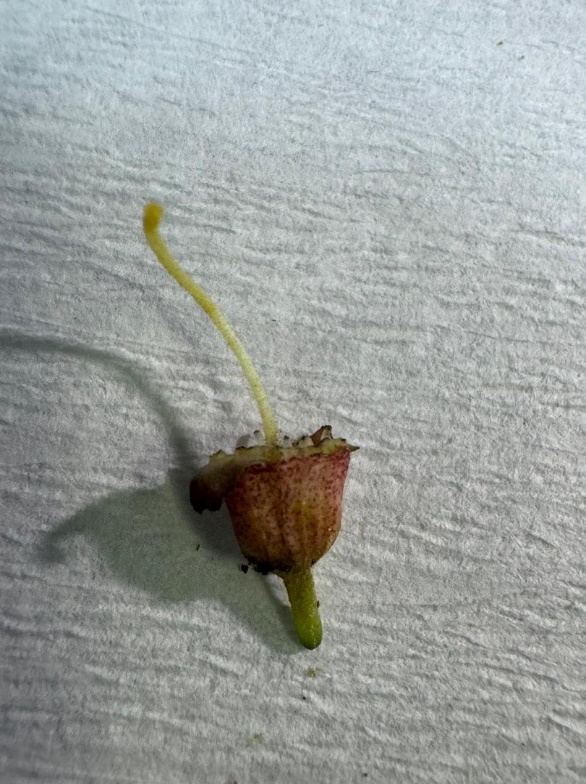** | **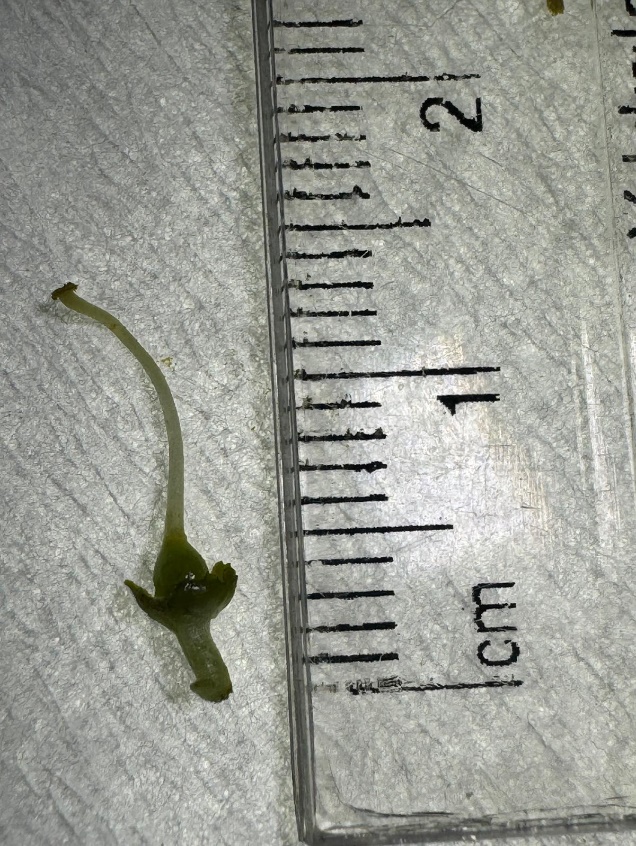**  **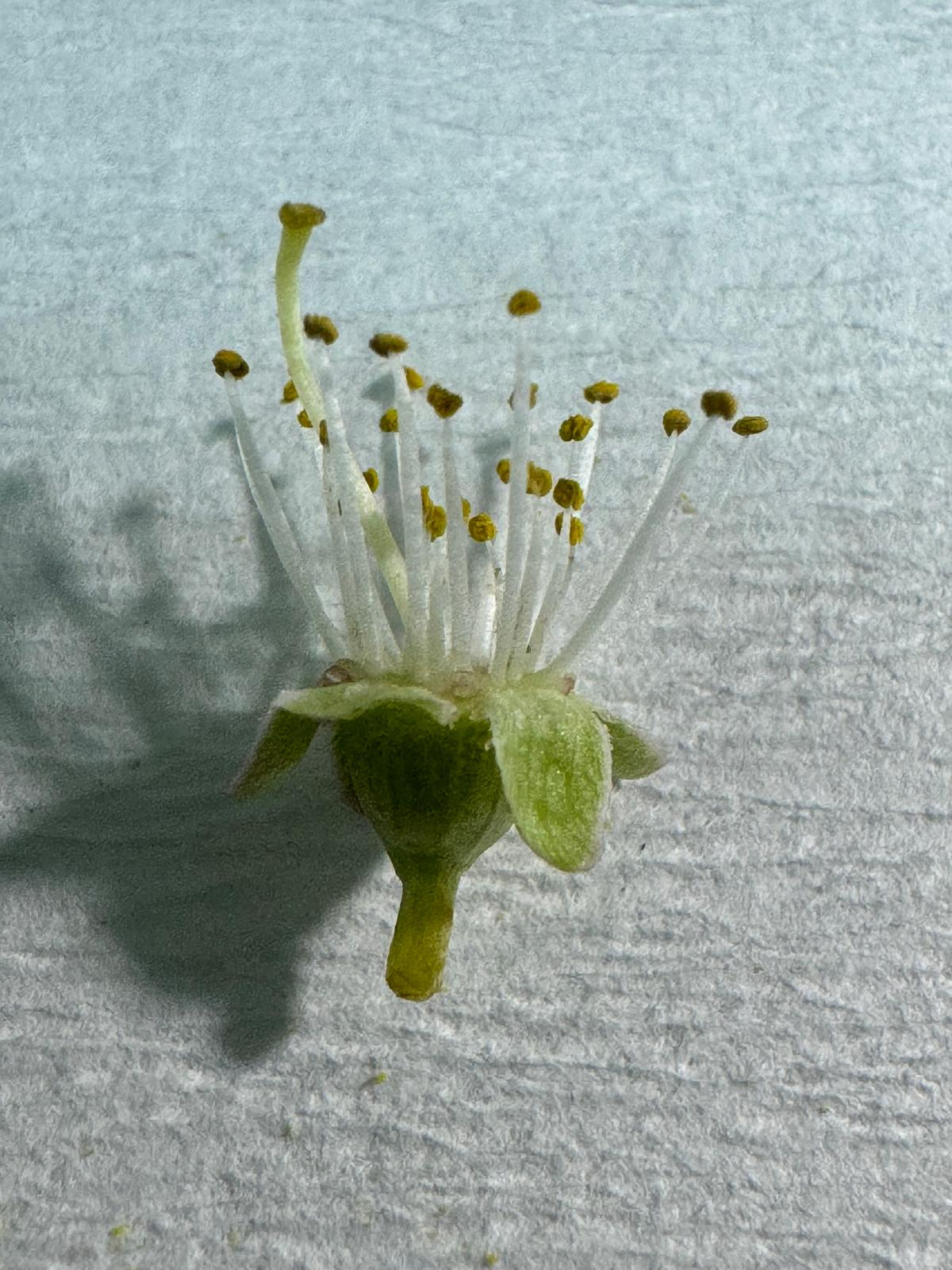** | **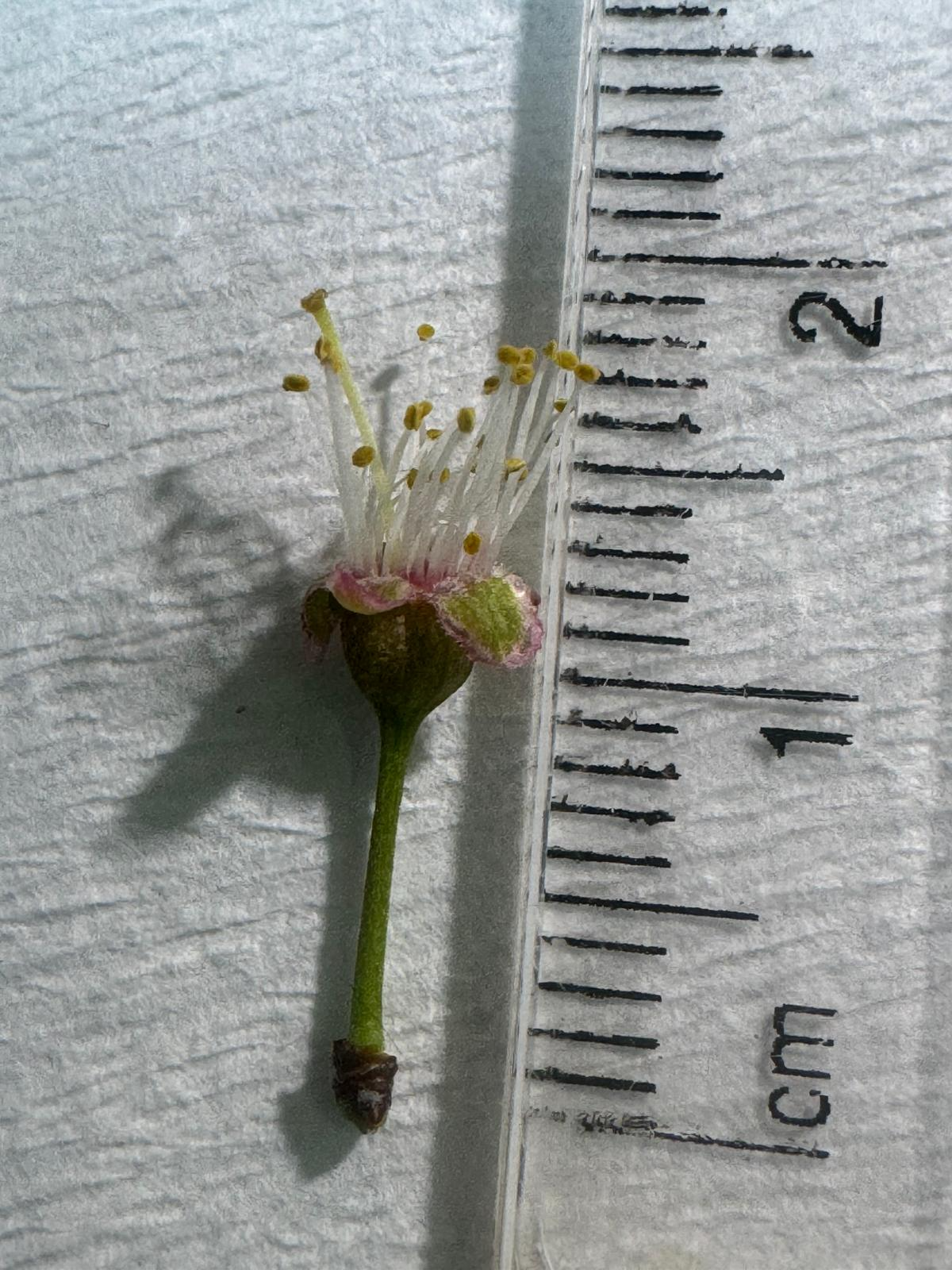**  **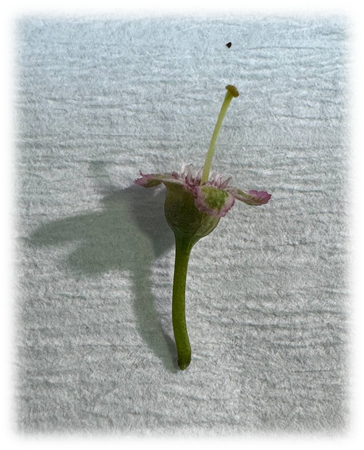** |
| Carpel characteristics | | |
| Yellow, short, medium size | Large, short, thick, deep green | Long, thin, green |
| Pedicel length (cm) | | |
| 0.88 ± 0.10 a | 0.32 ± 0.06 c | 0.35 ± 0.05 b |

This photograph was captured as part of the present study and was provided by the second author, Prof. Dr. Kahraman Gürcan. It is not subject to any third-party copyright restrictions.

**Supplementary Table S4.** Morphological comparison of anther traits in the local apricot cultivar ‘Kayseri Pa’, *Prunus* spp., and the control plum ‘Papaz Eriği’.

| Apricot | *Prunus* spp. | Plum |
| --- | --- | --- |
| **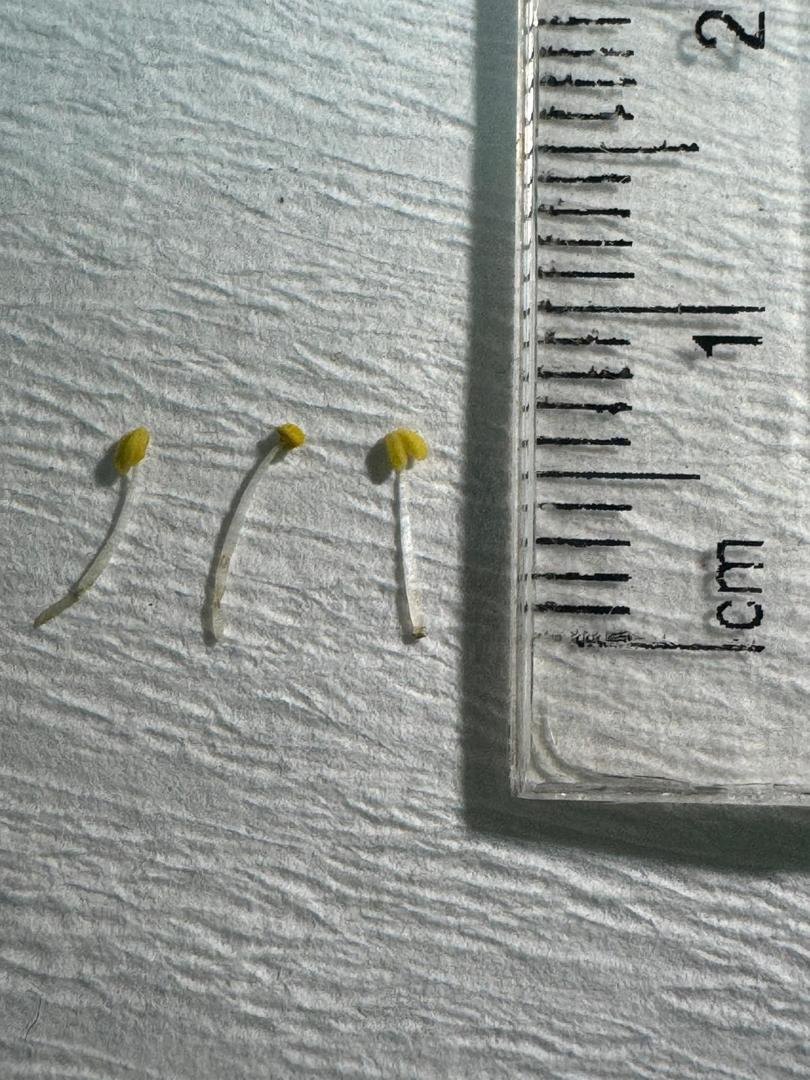**  **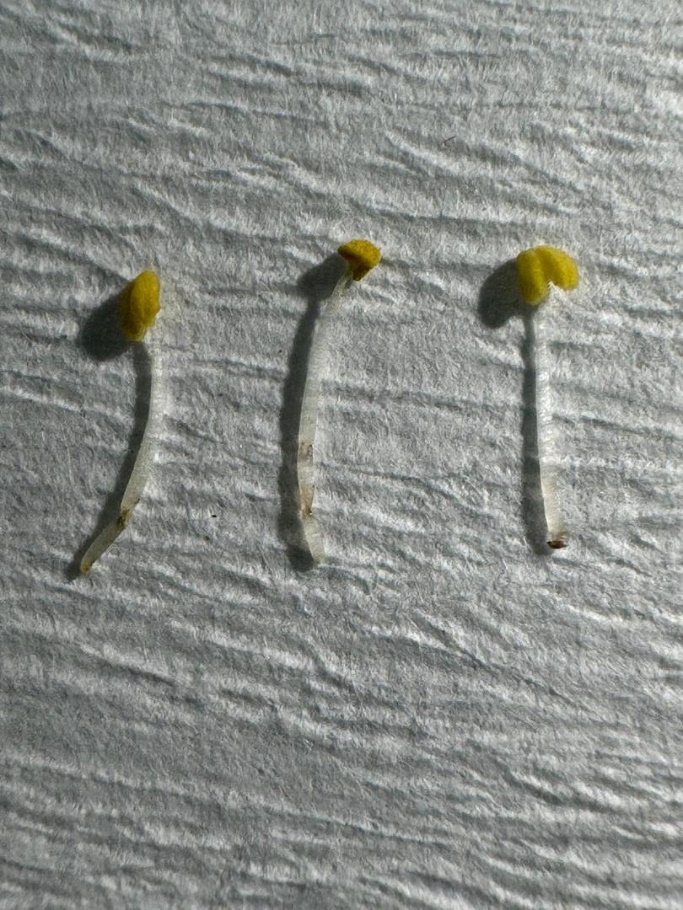** | **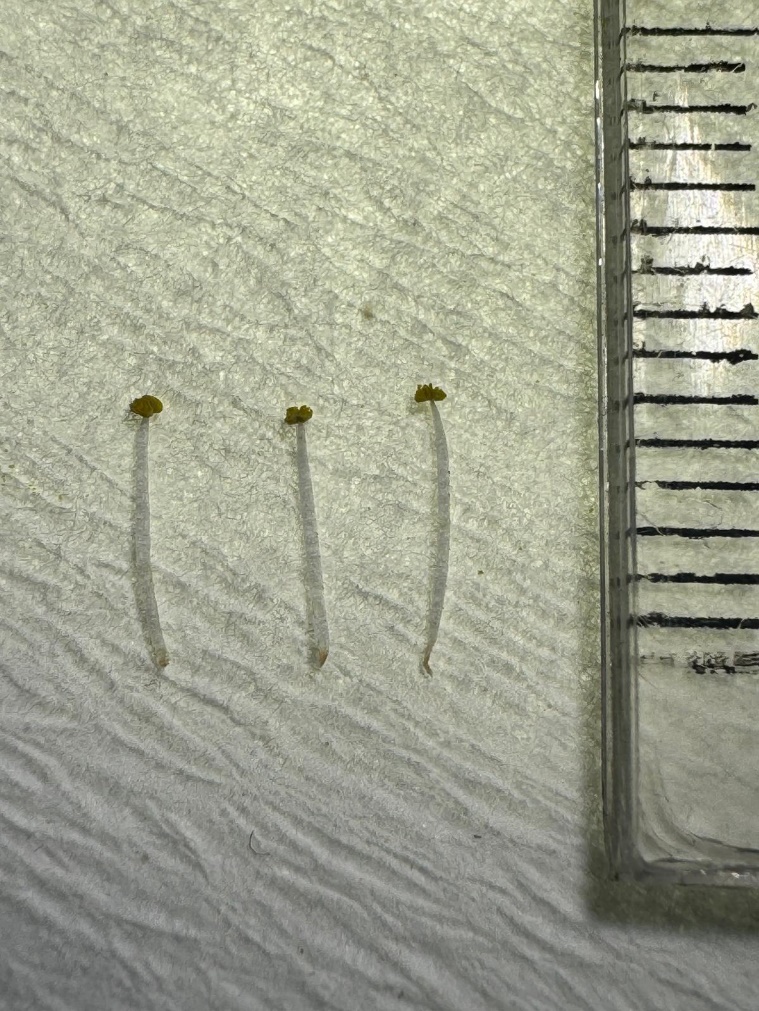**  **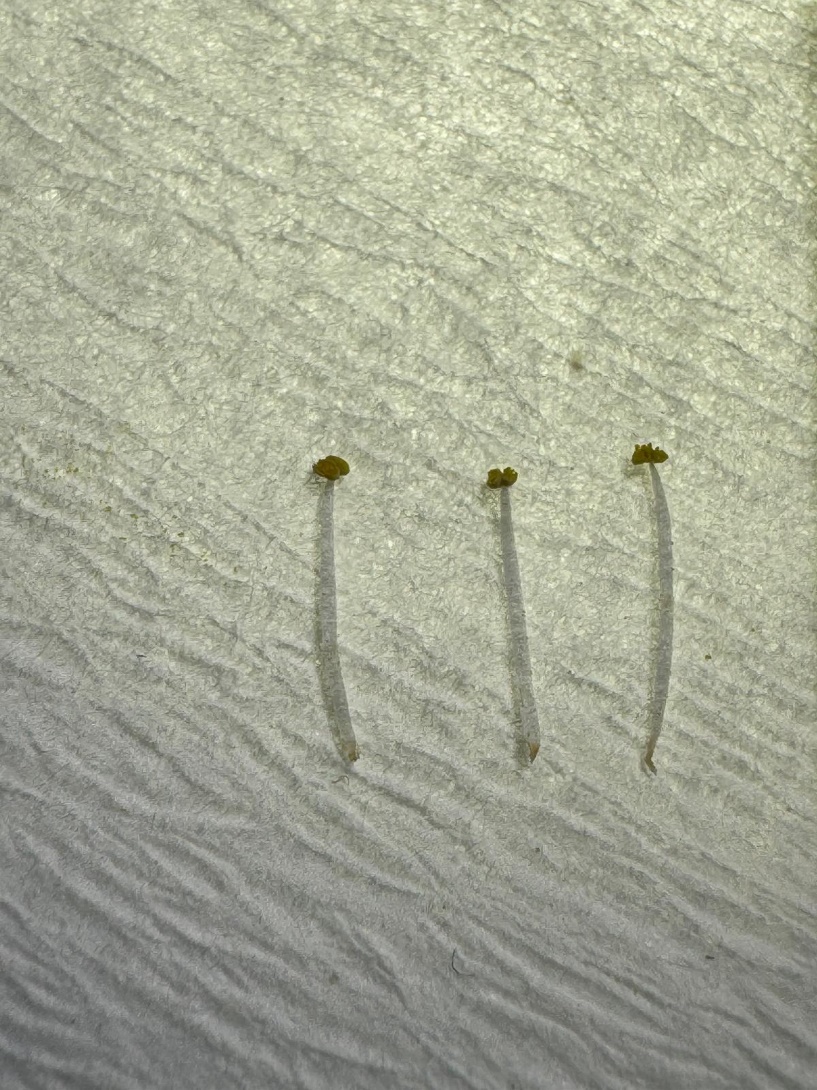** | **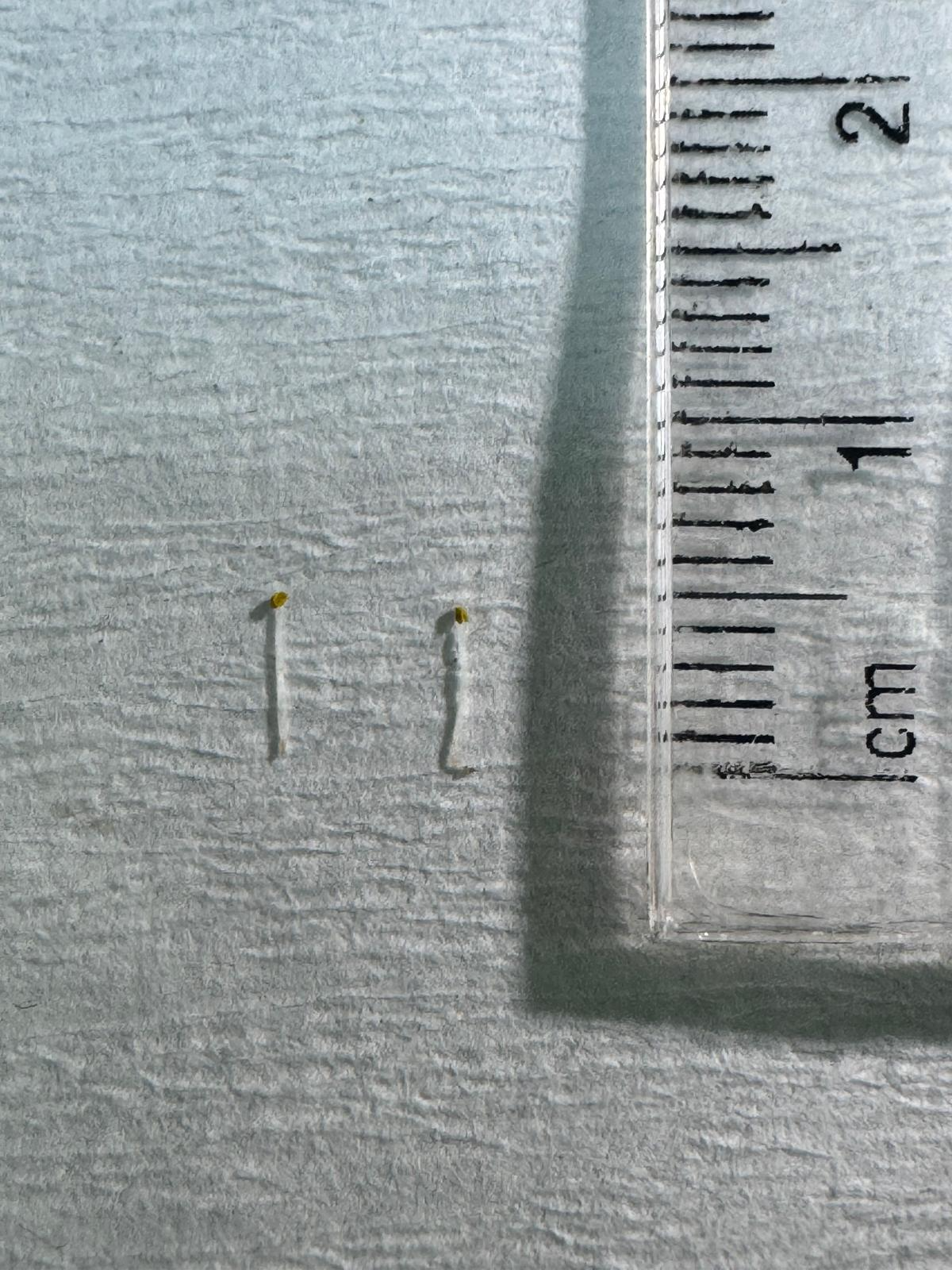**  **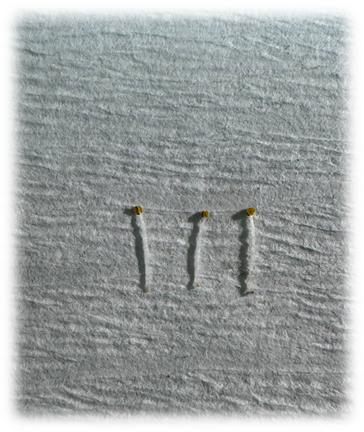** |
| Anther characteristics | | |
| Large yellow anthers with thick filaments | Small yellow anthers with thin filaments | Medium anthers with medium filaments |
| Filament length (cm) | | |
| 0.50 ± 0.04 c | 0.61 ± 0.02 a | 0.60 ± 0.08 b |
| Stamens per flower | | |
| 22.00 ± 0.82 c | 23.50 ± 0.58 a | 22.25 ± 0.96 b |

This photograph was captured as part of the present study and was provided by the second author, Prof. Dr. Kahraman Gürcan. It is not subject to any third-party copyright restrictions.

**Supplementary Table S5.** Petal characteristics of the local apricot cultivar ‘Kayseri Pa’, *Prunus* spp., and the control plum ‘Papaz Eriği’.

| Apricot | *Prunus* spp. | Plum |
| --- | --- | --- |
| **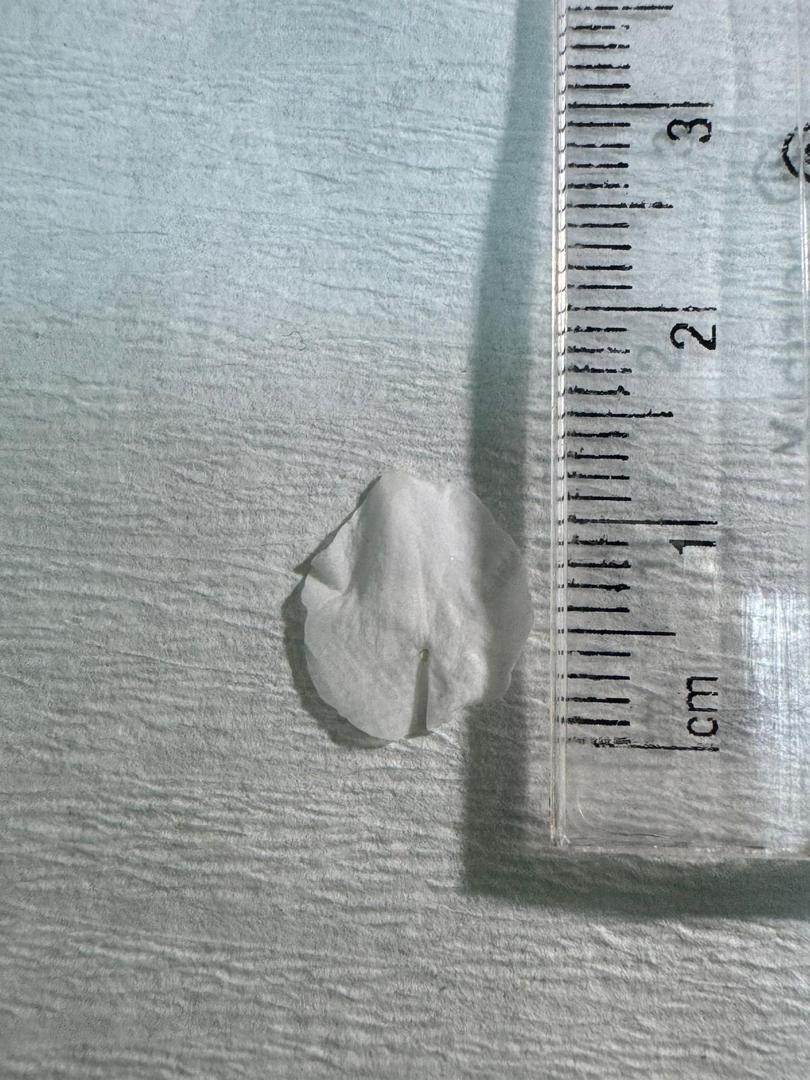**  **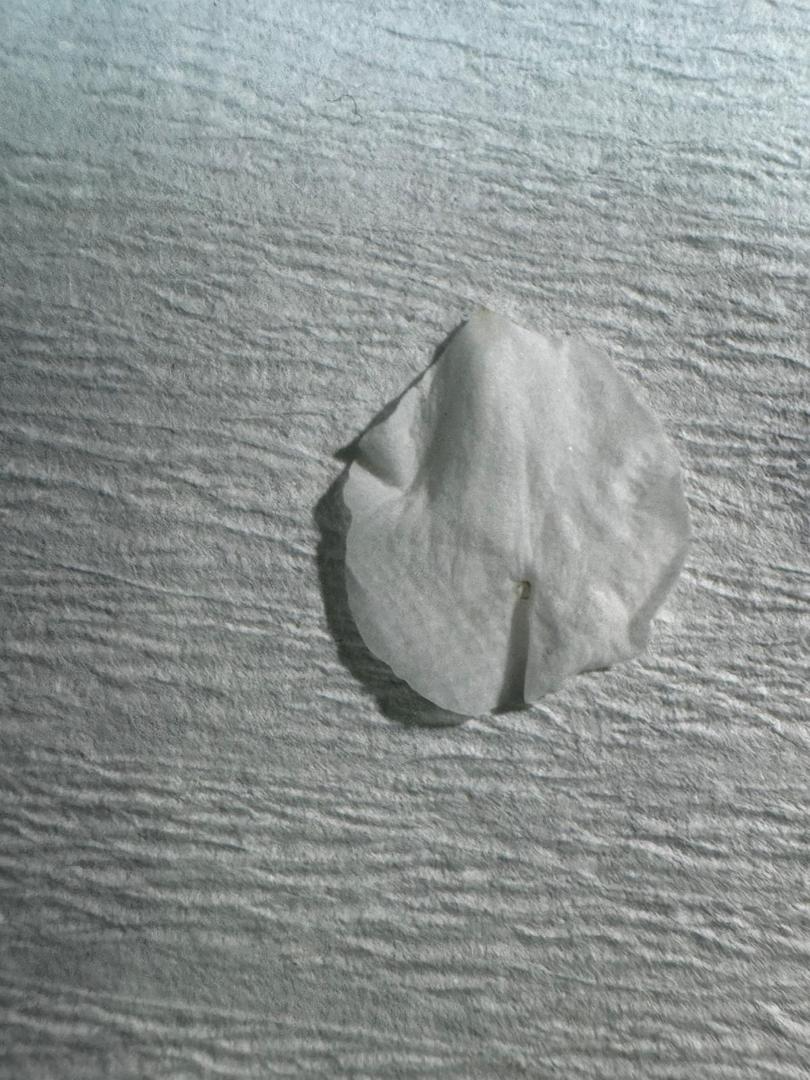** | **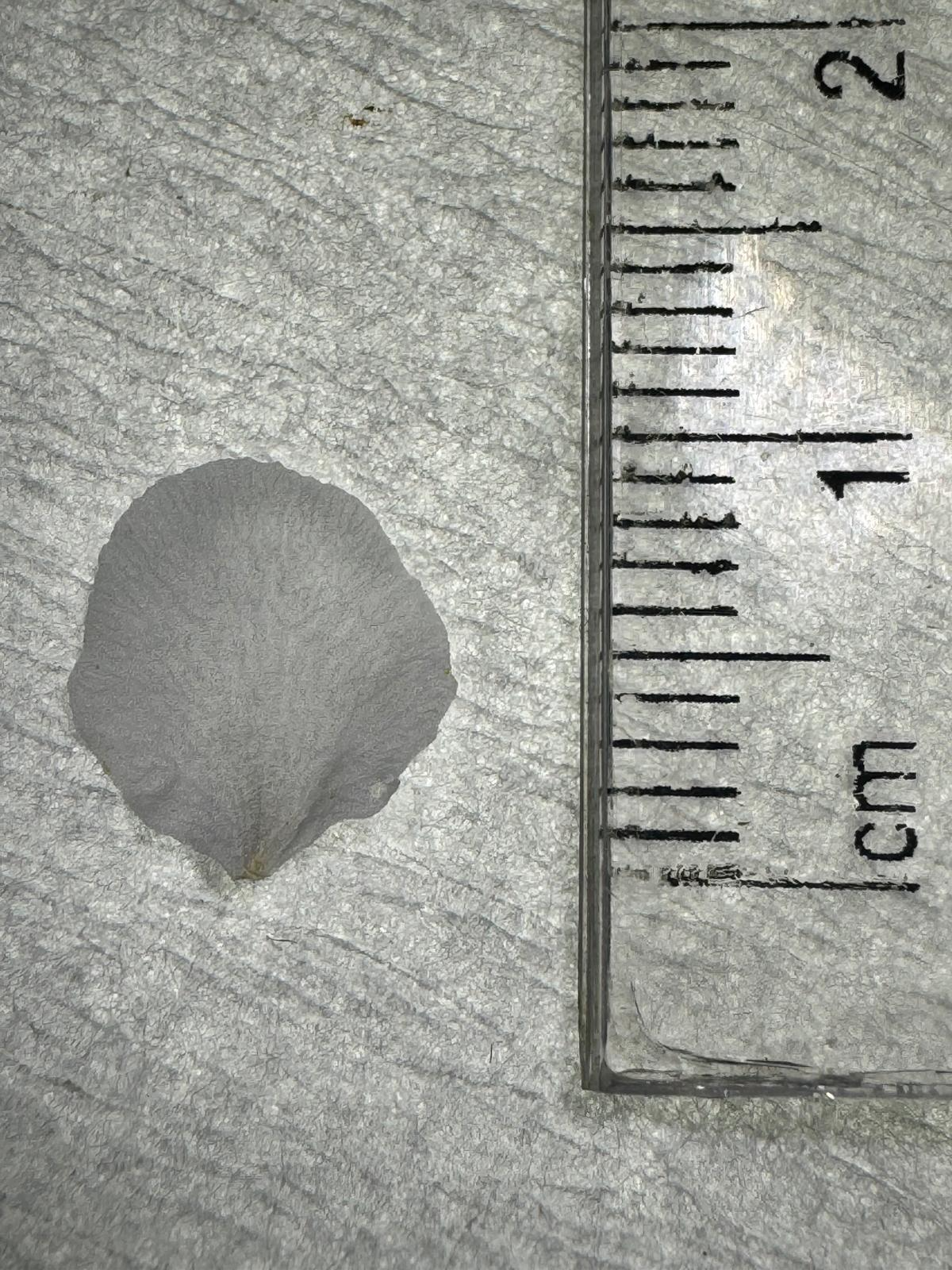**  **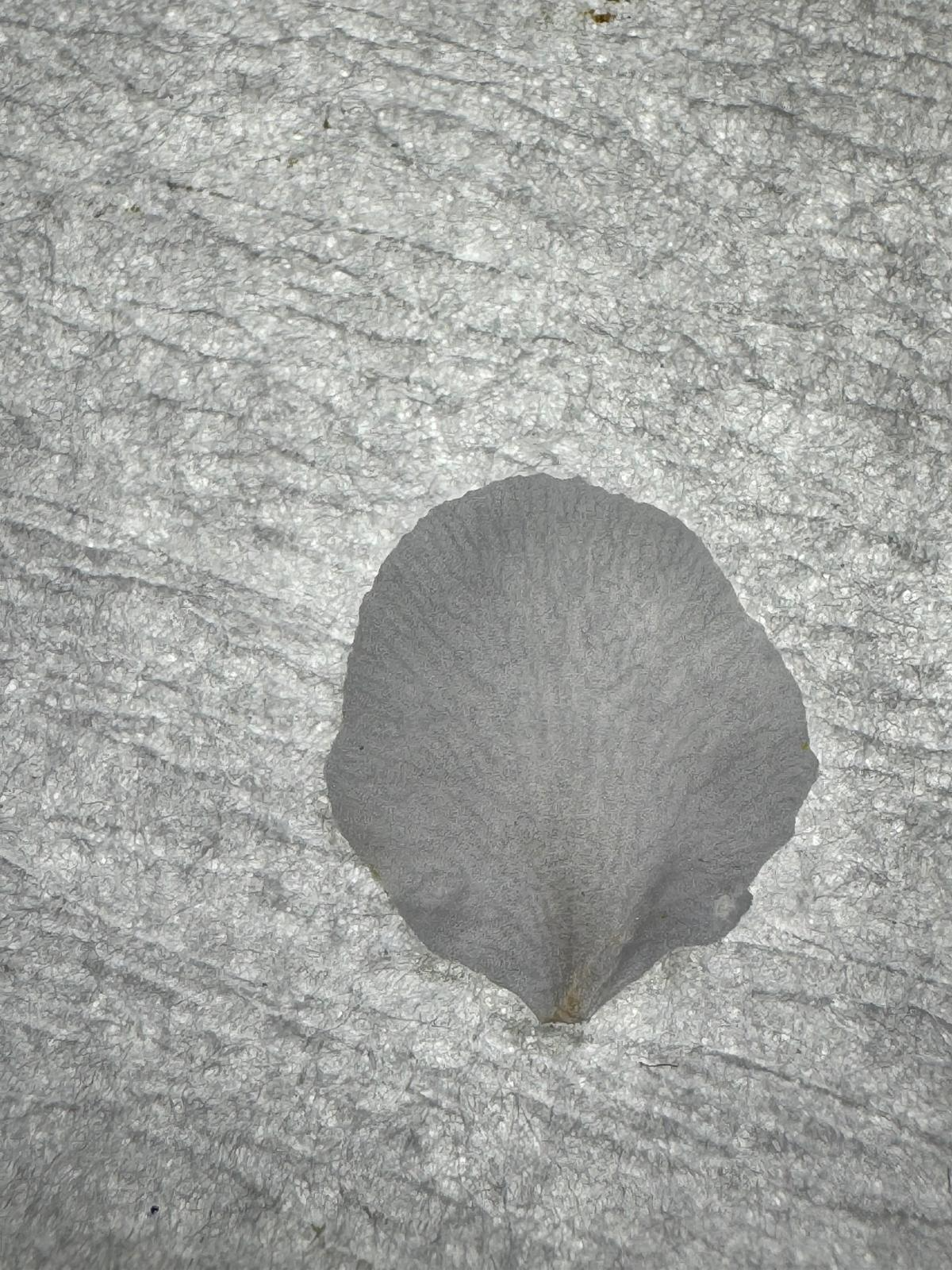** | **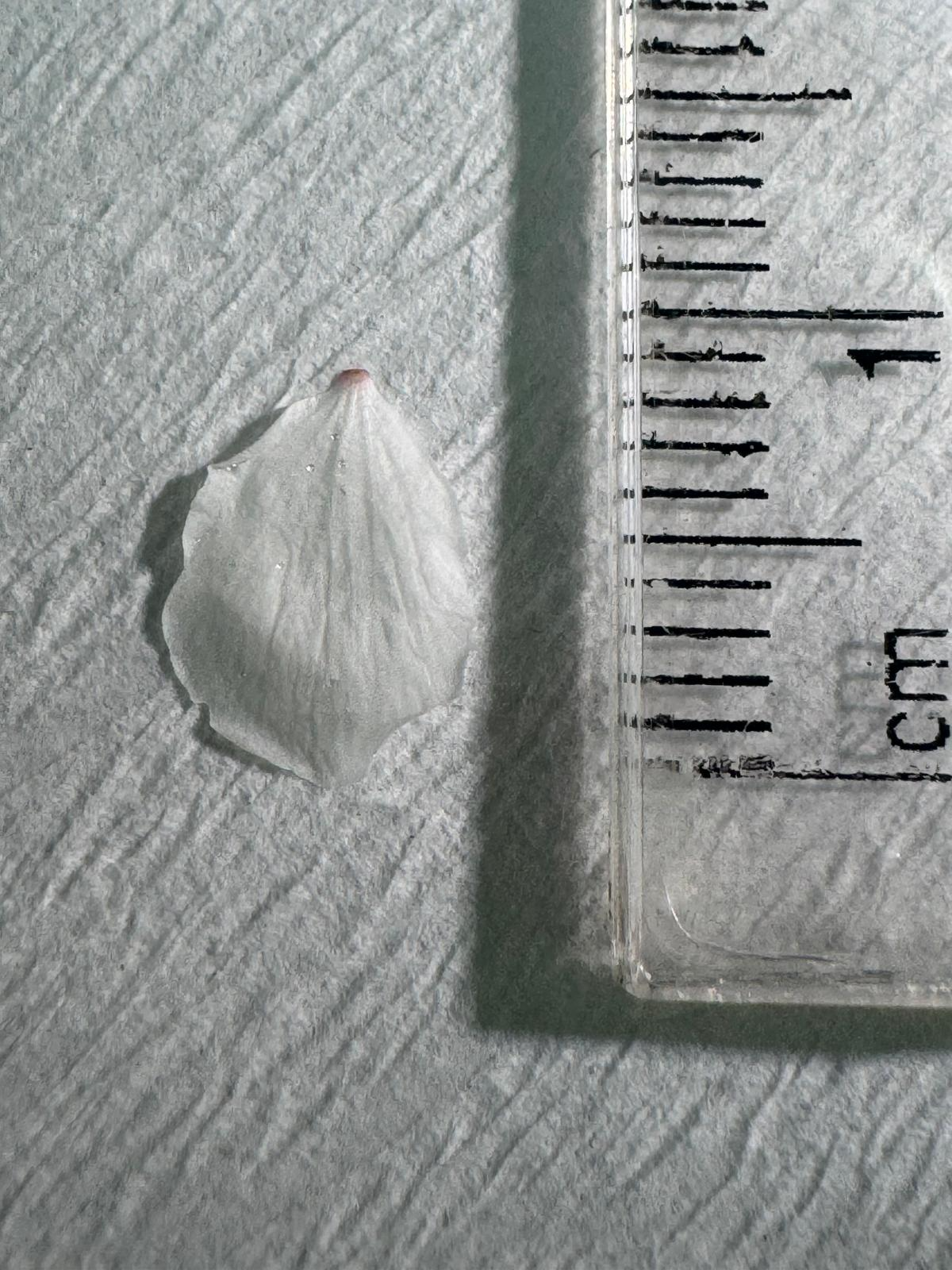**  **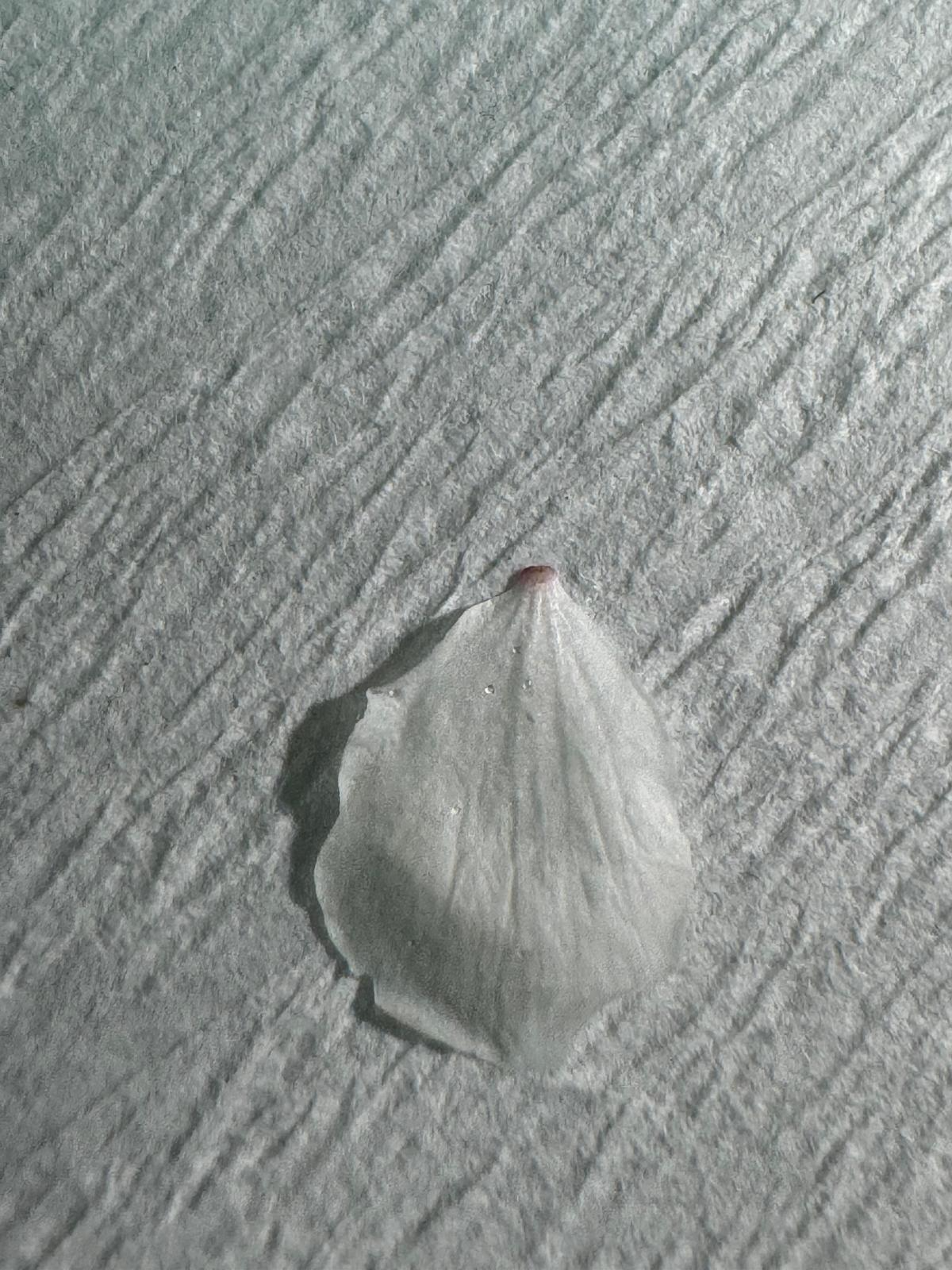** |
| Petal characteristics | | |
| long, medium width | wide, medium length | medium length and width |
| Petal length (cm) | | |
| 1.2 | 1.0 | 0.9 |

This photograph was captured as part of the present study and was provided by the second author, Prof. Dr. Kahraman Gürcan. It is not subject to any third-party copyright restrictions.

**Supplementary Table S6.** Gene content and functional classification of the plastome of wild plum (*Prunus* spp.) from the Erciyes Mountain population.

| | **Category** | **Gene group** | **Gene names** | | --- | --- | --- | | **Self-replication**  **Photosynthesis**  **Other genes**  **Unkown** | Large subunit of ribosome  Small subunit of ribosome  DNA dependent RNA polymerase  rRNA genes  tRNA genes  Photosystem I  Photosystem II  ATP synthase  NADH-dehydrogenase  Cytochrome b/f complex  Rubisco  Acetyl-CoA-carboxylase  c-type cytochrom synthesis gene  Envelop membrane protein  Protease  Maturase  Conserved open reading frames | rpl12, 14, 16, 20, 22, 23, 32, 33.  rps 3, 4, 7*, 8, 11, 12* 14,15,16,18,19*  rpoA, B, C1, C2  rrn16, 23, 4.5, 5  trnH-GUG, trnK-UUU, trnQ-UUG, trnS-GCU, trnS-CGA, trnR-UCU, trnC-GCA, trnD-GUC, trnY-GUA, trnE-UUC, trnT-GGU, trnS-UGA, trnG-GCC, trnM-CAU, trnS-GGA, trnT-UGU, trnL-UAA, trnF-GAA, trnM-CAU, trnW-CCA, trnP-UGG, trnM-CAU, trnL-CAA, trnV-GAC, trnE-UUC, trnA-UGC, trnR-ACG, trnN-GUU, trnL-UAG, trnN-GUU, trnR-ACG, trnA-UGC, trnE-UUC, trnV-GAC, trnL-CAA, trnM-CAU  psaA, B, C, I, J  psbA, B, C, D, E, F, I, J, K, L, M, N,T, Z  atpA, B, E, F, H, I  ndhA, B*, C, D, E, F, G, H, I, J, K  petA, B, D, G, N  rbcL  accD  ccsA  cemA  clpP  matK  ycf2, ycf3, ycf4 |   * indicates duplicated genes located in the inverted repeat (IR) regions. |  |  |  |  |  |  |  |  |
| --- | --- | --- | --- | --- | --- | --- | --- | --- | --- | --- | --- | --- | --- | --- |

**Supplementary Table S7.** Structural features of the plastome of wild plum (*Prunus* spp.) from the Erciyes Mountain population.

| **Species** | **Length (bp)** | **LSC (bp)** | **SSC (bp)** | **IRa (bp)** | **IRb (bp)** | **GC content (%)** | **Total genes** | **tRNA genes** | **rRNA genes** |
| --- | --- | --- | --- | --- | --- | --- | --- | --- | --- |
| Wild plum (Erciyes) | 157,840 | 86147-157840 | 112524-131463 | 131464 - 157840 | 86148 - 112525 | 36.8% | 125 | 36 | 8 |

LSC: large single-copy region; SSC: small single-copy region; IR: inverted repeat.


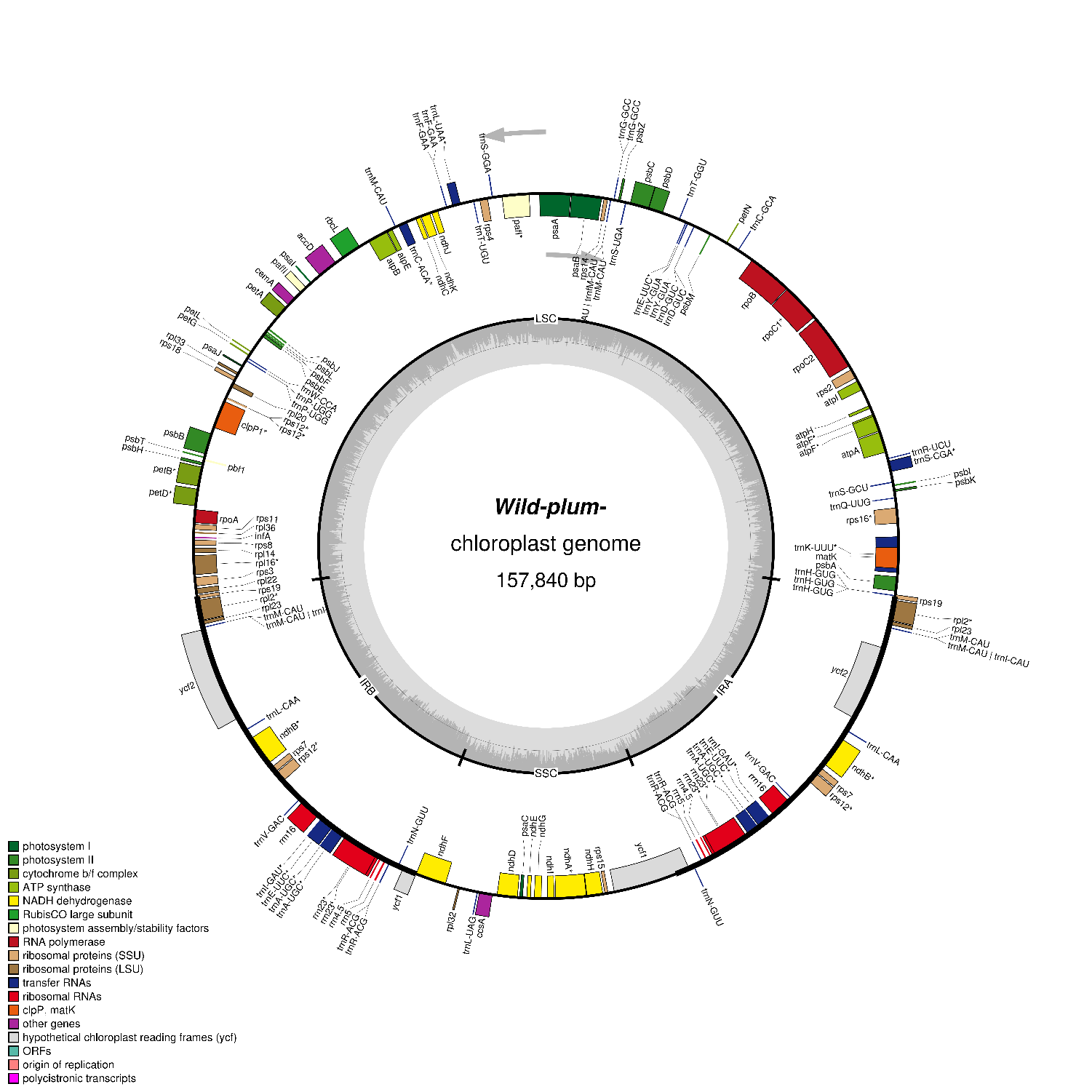


**Supplementary Fig. S1.** Circular map of the plastome of wild plum (*Prunus* spp.) from the Erciyes Mountain population. Genes located outside the circle are transcribed clockwise, whereas those inside are transcribed counterclockwise. Functional gene groups are color-coded as indicated in the legend. LSC, large single-copy region; SSC, small single-copy region; IR, inverted repeat.
